# Effects of operational taxonomic unit inference methods on soil microeukaryote community analysis using long‐read metabarcoding

**DOI:** 10.1002/ece3.8676

**Published:** 2022-03-08

**Authors:** Shadi Eshghi Sahraei, Brendan Furneaux, Kerri Kluting, Mustafa Zakieh, Håkan Rydin, Håkan Hytteborn, Anna Rosling

**Affiliations:** ^1^ Department of Ecology and Genetics Uppsala University Uppsala Sweden; ^2^ Department of Plant Breeding Swedish University of Agricultural Sciences Alnarp Sweden

**Keywords:** clustering, ITS, rDNA, species hypothesis

## Abstract

Long amplicon metabarcoding has opened the door for phylogenetic analysis of the largely unknown communities of microeukaryotes in soil. Here, we amplified and sequenced the ITS and LSU regions of the rDNA operon (around 1500 bp) from grassland soils using PacBio SMRT sequencing. We tested how three different methods for generation of operational taxonomic units (OTUs) effected estimated richness and identified taxa, and how well large‐scale ecological patterns associated with shifting environmental conditions were recovered in data from the three methods. The field site at Kungsängen Nature Reserve has drawn frequent visitors since Linnaeus's time, and its species rich vegetation includes the largest population of *Fritillaria meleagris* in Sweden. To test the effect of different OTU generation methods, we sampled soils across an abrupt moisture transition that divides the meadow community into a *Carex acuta* dominated plant community with low species richness in the wetter part, which is visually distinct from the mesic‐dry part that has a species rich grass‐dominated plant community including a high frequency of *F*. *meleagris*. We used the moisture and plant community transition as a framework to investigate how detected belowground microeukaryotic community composition was influenced by OTU generation methods. Soil communities in both moisture regimes were dominated by protists, a large fraction of which were taxonomically assigned to Ciliophora (Alveolata) while 30%–40% of all reads were assigned to kingdom Fungi. Ecological patterns were consistently recovered irrespective of OTU generation method used. However, different methods strongly affect richness estimates and the taxonomic and phylogenetic resolution of the characterized community with implications for how well members of the microeukaryotic communities can be recognized in the data.

## Graphical abstract

We use phylogenetic analysis to compare the effect of three different sequence clustering methods applied to long environmental rDNA amplicon sequences of soil microeukaryotic communities. We demonstrate that different clustering methods have a strong effect on estimated richness and identified taxa, but all methods recover large scale ecological patterns associated with shifting meadow plant diversity across a soil moisture transition.

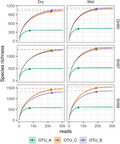

## 1 INTRODUCTION

Microbial community composition in soil can be assessed in metabarcoding studies of environmental DNA (eDNA) extracts by amplification and sequencing of barcoding regions, often targeting the ribosomal operon. Richness estimates based on eDNA metabarcoding studies indicate that global fungal species richness is at least ten times higher than the number of formally described species (Spatafora et al., 2017), including several class level lineages of currently undescribed fungi (Tedersoo et al., 2017). Non‐fungal microeukaryotes, collectively referred to as protists throughout the text, are far less studied in soil compared with fungi but are increasingly recognized for their diverse ecosystem functions (Geisen, 2016). Recent molecular studies using eDNA have dramatically increased our knowledge of protist diversity in different environments, even indicating that diversity may be higher in soil than in water (Burki et al., 2021; Geisen et al., 2018; Mahé et al., 2017).

Challenges in characterizing soil microeukaryotic communities from metabarcoding data include biases associated with primer choice, tradeoffs between number of samples and sequencing depth, method for estimating species richness as well as accuracy of taxonomic identification of community members. Some of these aspects are discussed below and further explored in this paper. The two internal transcribed spacer (ITS1 and ITS2) are noncoding, hypervariable regions of the rDNA operon, widely accepted as marker regions for characterization of fungal communities (Schoch et al., 2012). However, due to intraspecific variation and sequencing errors, community composition of known and novel species cannot be directly identified from the massive numbers of unique reads generated by high‐throughput eDNA sequencing (Ryberg, 2015). Instead, sequence reads are clustered into operational taxonomic units (OTUs) and/or denoised into amplicon sequence variants (ASVs) that may serve as proxies for species. In a comparison of OTU clustering and denoising into ASVs of short‐read amplicon, Glassman and Martiny (2018) demonstrated that the two methods capture different representations of the soil microeukaryotic community but that large‐scale ecological patterns were consistently represented in both datasets. Similarly, spatio‐temporal turnover patterns were consistently captured across datasets using both different sequencing technologies and different amplicon lengths (Furneaux et al., 2021). While strong community patters are highly reproducible, effects of bioinformatic tools on the generated species proxies and the sequences selected to represent them remains important for researchers that which to take metabarcoding community analysis beyond large‐scale ecological patterns.

The most common approach for OTU generation has been abundance‐based greedy clustering of reads using fixed similarity thresholds relative to a centroid sequence, as implemented in USEARCH (Edgar, 2013) and VSEARCH (Rognes et al., 2016). Clustering thresholds are often chosen based on estimates of the level of variation present within species (Tedersoo et al., 2014). However, no universal threshold accurately separates all species (Nilsson et al., 2008; Vu et al., 2019), and a more stringent threshold may cause two sequences which belong to the same species to separate into different OTUs, that is, splitting of species, while a less stringent threshold may artificially lump multiple species together into a single OTU (Ryberg, 2015). In single‐linkage clustering on the other hand, a read is joined to a cluster if it is within the set similarity threshold to any other read in the cluster, that is. not just compared with a centroid sequence. This approach has been used with similarity thresholds much smaller than the expected sequencing error (e.g., 1 bp) to delimit more “natural” OTUs, as applied in swarm clustering (Mahé et al., 2014). Very small similarity thresholds are only appropriate in a densely populated error space, and the presence of intermediate sequences can cause single‐linkage clustering to group fairly distant sequences into an OTU (Mahé et al., 2014, 2017). Clustering based on similarity thresholds, whether centroid‐based or single‐linkage, does not differentiate sequencing errors from biological variation. Denoising algorithms, such as DADA2, have been developed to identify ASVs present in a sample, by removing sequencing errors using a model which incorporates the base quality scores and read abundances (Callahan et al., 2016). This approach captures both within and between species variation, even as little as one base pair difference, and so ASVs may be further clustered to serve as proxies for species (Frøslev et al., 2017). However, DADA2 does rely on the presence of at least two identical sequences as seeds for generating ASVs, so the method can perform poorly when the majority of reads are singletons (Furneaux et al., 2021).

Assigning taxonomy to OTUs may allow for functional analysis of community composition, but is highly dependent on curated reference datasets such as the PR2 for protists (Del Campo et al., 2018; Guillou et al., 2012) and UNITE for fungi (Kõljalg et al., 2013). In the well‐established fungal sequence database UNITE, OTUs are derived using a range of thresholds from 97% to 99.5% similarity across the ITS2 region and referred to as species hypotheses (SH) with unique numbers and known species names when available (Kõljalg et al., 2013). The development of PacBio sequencing technology (Pacific Biosciences, Menlo Park, CA, SA) has allowed longer eDNA amplicons, including both variable spacers and more conserved functional rDNA regions, to be sequenced from complex samples. In the absence of matching reference sequences, taxonomic assignment of novel lineages is possible based on phylogenetic inference using the more conserved rDNA small subunit (SSU; Jamy et al., 2020) and/or large subunit (LSU) sequences (Furneaux et al., 2021; Tedersoo et al., 2017). The benefit of phylogenetically supported taxonomic assignment of OTUs is particularly relevant in communities consisting mostly of poorly characterized lineages (Kalsoom Khan et al., 2020).

For this study, we revisited two permanent transects at the Kungsängen Nature Reserve (Sernander, 1948; Zhang, 1983). Kungsängen is a seminatural grassland located in Uppsala, Sweden, home to a large population of the plant *Fritillaria meleagris* (Liliaceae) in Sweden, where it was naturalized in the 18^th^ century after being used as a popular garden flower since the 17th century (Linnaeus, 1921 [1753]; Zhang, 1983). At the site we collected plant community data across the abrupt change in meadow plant community from the wetter part towards the river, visually distinct from the mesic‐dry part further inland. Soil samples were collected on both sides of this transition zone, with and without *F*. *meleagris*, for the first belowground community observations from this study site. More importantly, we tested if belowground community compositional shift across the transition from wet to mesic‐dry parts of the meadow was consistently captured with different OTU generation methods. Further, the effect of OTU generation method on the characterized community of soil microeukaryotes was explored for richness estimates, taxonomic and phylogenetic resolution and detection limits of rare taxa. While long‐read metabarcoding is becoming an increasingly popular methodology in eDNA community analysis (Burki et al., 2021; Furneaux et al., 2021; Jamy et al., 2020; Leho Tedersoo et al., 2017) available bioinformatic tools for sequence clustering are primarily developed and tested for short‐read amplicon datasets. Our study addresses this knowledge gap by outlining a phylogenetic context for analyzing differences in soil microeukaryotic communities captured with three different sequence clustering methods applied to the same long‐read amplicon dataset.

## 2 MATERIAL AND METHODS

### 2.1 Field site

**FIGURE 1:**
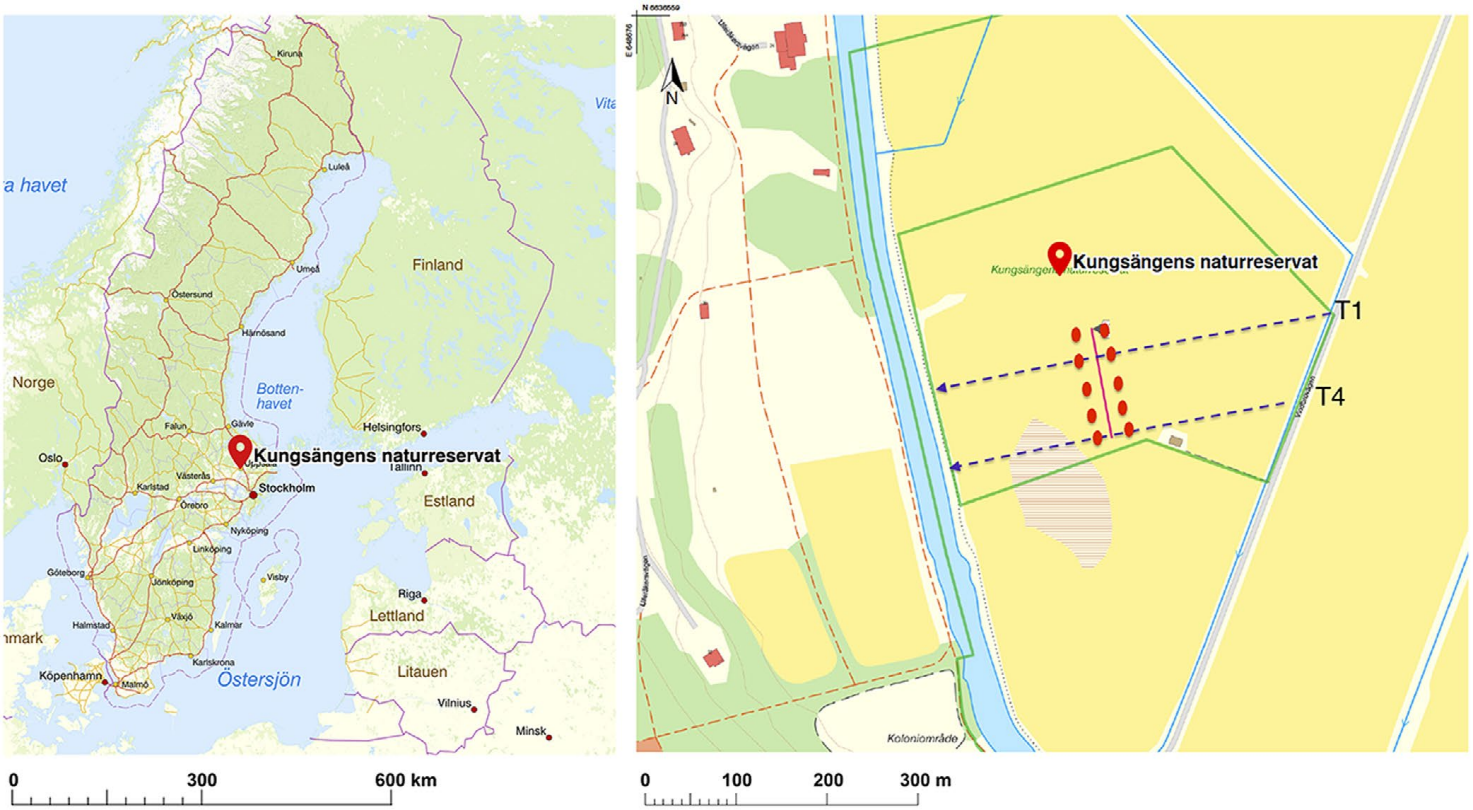
The Kungsängen Nature Reserve field site is located. (a) south of the city of Uppsala, in central Sweden (red circle). (b) It is part of a large meadow on the east side of the Fyris River, a green line indicating reserve borders. Red dots indicate soil sampling locations on either side of the soil moisture transition border (red line) intersecting two permanent plant community transects T1 and T4 (dashed lines). Map source: © Lantmäteriet, i2012/921

Kungsängen Nature Reserve (N59°50’, E17°40’) is a 12.5‐hectare reserve in a larger meadow located in the south of Uppsala, Sweden, along the east shore of the Fyris River (Figure 1). The eastern mesic‐dry part of the meadow is managed by annual hay making in late July, while the western part is managed less frequently because of high soil moisture due to its low elevation and proximity to the Fyris River (Zhang, 1983; Zhang & Hytteborn, 1985). To investigate vegetation in the field, 28 permanent plots (2 × 2 m) were laid out across an east–west transect in the meadow in the 1940s (Sernander, 1948). Along this transect 1, plots are located from 1.07 and 2.57 m above sea level. In the 1980s, three additional parallel transects (2–4) were laid out (Zhang, 1983).

In 2016–2017, two of the four permanent transects (1: plots 1–28 in June 2016 and 4: plots 61–76 in June 2017) were revisited, and the plant community was inventoried using the 5‐degree Hult‐Sernander‐Du Rietz logarithmic scale (5, 50%–100% cover; 4, 25%–50%; 3, 12.5%–25%; 2, 6.25%–12.5%; 1, <6.25%). The transects spans the length of the meadow from the river in the west to the edge of the reserve in the east. Although sampling plots are not marked in the terrain, the starting point of the transect, its direction, and distance between plots is very well documented in the original publication (Sernander, 1948). Members in our team were involved in subsequent inventories of the meadow performed in the 1980s (Zhang, 1983), ensuring that the locations of the sampling plots are within a few meters of the original plots.

### 2.2 Soil sampling

Soil samples were collected to test for compositional differences in the soil microeukaryotic community in response to both large‐scale environmental differences, that is, wet versus mesic dry soil conditions, and small‐scale habitat differences, that is, presence or absence of a *F*.* meleagris* plant. A visual vegetation shift marks the soil moisture transition from the wet area to the mesic‐dry area further inland. The soil type at the site ranges from light clay with fine sand to heavy clay, with a higher clay content in the wet compared with the mesic dry part of the meadow (Zhang, 1983). This transition border falls close to plot 16 on transect 1 and plot 68 on transect 4 (red line in Figure 1). Soil samples were collected on June 7th, 2016, from five locations 30 m apart on each side of the soil moisture transition border separated by 30 m across the transition border. Soil sampling locations intersected transect 1 and 4 between plots 14 and 67 in the mesic‐dry and plots 17 and 69 in wet area (Figure 1). At each location, two soil samples were taken using a soil corer when possible (5 cm diameter × 10 cm depth) or with a hand shovel when soils were too wet for using a soil corer (as was the case for most of the samples on the wet side). When using a shovel, sampling depth and soil volume were estimated to correspond to that of the soil cores. The first sample at each location was collected around a *F*. *meleagris* plant and the second at 0.5 m distance from the sampled *F*. *meleagris* plant. For the second sample (referred to as non‐*Fritillaria* soil), we also ensured that no other *F*. *meleagris* plant was within 0.5 m of the sample. The corer/shovel was wiped with 70% ethanol‐soaked tissue paper between each sampling. In total, 20 soil samples were collected: five *Fritillaria*/non‐*Fritillaria* soil sample pairs from the wet side and five pairs from the mesic‐dry side. The most common plant species were recorded at each sampling location for cross reference to the more complete plant community data recorded for plots along the transects (Table S1).

All samples were individually placed in plastic bags and kept on ice during sampling before storage at 4°C overnight. The following day, soils were homogenized in the plastic bags and subsamples of soil were transferred to 15 ml conical centrifuge tubes and frozen at −20°C, followed by freeze drying. Another subsample was weighed before drying at +80°C for 48 h to estimate gravimetric soil moisture (Holliday, 1990; Table S2).

### 2.3 Library preparation and sequencing

Around 300 mg of freeze‐dried soil was used for total DNA extraction using a NucleoSpin^®^Soil kit (Macherey‐Nagel). DNA concentration and purity of extracts were measured using a NanoDrop 2000 (Thermo Fisher Science), and concentrations ranged from 90 to 320 ng/µl. The entire ITS and partial LSU regions of the rDNA operon were amplified using the forward primer ITS1 (5′–TCCGTAGGTGAACCTGC–3´) modified by removing the two GG nucleotides from the 3´end compared with the original ITS1 primer (White et al., 1990), and reverse primer LR5 (5′–TCCTGAGGGAAACTTCG–3’) (Vilgalys & Hester, 1990). These primers were selected because they amplify most known fungi (Tedersoo et al., 2015) and had no known mismatches to most available sequences in Glomeromycota (Krüger et al., 2012). In addition, the primers capture a wide range of non‐fungal microeukaryotes. Barcodes added to forward and reverse primers were combined in sample‐specific barcode pairs for multiplexed sequencing (Table S3). Each 40 µl PCR reaction contained 20.4 µl nuclease free water, 0.4 µl Phusion High‐Fidelity DNA Polymerase (Thermo Fisher Scientific, Hudson, NH, US), 8 µl 5× buffer, 0.01 µM of each primer, 200 µM dNTP mix, 2 µl DMSO, and 4 µl DNA template. The thermal cycle protocol used was a 10 min initial denaturation at 95°C, 25 cycles of denaturation (45 s, 95°C), annealing (45 s, 59°C) and elongation (90 s, 72°C), and a final elongation (72°C, 10 min). PCR products were visualized by gel electrophoresis. The resulting amplicons (approximately 1500 bp long) were purified with the ZR‐96 DNA clean up kit (Zymo Research). The amount of PCR products used for the pooled library was estimated to approximate equimolar amounts based on observed electrophoresis band intensity. The pooled library was sequenced together with root samples from the site at SciLifeLab/NGI (Uppsala, Sweden) with six SMRT cells on the PacBio RSII sequencing platform. Raw demultiplexed reads for the current study are available in ENA (accession number: PRJEB47280).

### 2.4 Bioinformatic analysis

RSII subread files in BAX format were converted to the newer BAM format using “bax2bam” from PacBio SMRT tools 5.0.1, and reads were demultiplexed using “lima” from PacBio SMRT tools 7.0.1 using the options “‐‐different” and “‐‐peek‐guess”. Sequences which were not assigned to one of the barcode pairs used in this experiment were discarded. Circular consensus sequences (CCS) were generated from the demultiplexed BAM files using “ccs” from PacBio SMRT tools 5.0.1 (the last version which supports RSII data), resulting in 49,709 reads. Sequences were oriented in the forward direction by matching the forward and reverse primer sequences using Cutadapt v.3.0 (Martin, 2011) retaining only reads with both a forward and a reverse primer sequence in the correct orientation (ITS1 and reverse‐complemented LR5). Further, concatamers (Griffith et al., 2018) were identified by searching for the primer sequence pairs ITS1/reverse‐complemented ITS1, LR5/reverse‐complemented LR5, ITS1/ITS1, and LR5/LR5 within the forward and reverse strands of each of the reads, and if detected, the read was discarded. Remaining reads were length and quality filtered, allowing for read lengths of 50–2999 bp and a maximum of 12 expected errors per read (to account for the probability of errors accumulating over a long fragment), the AmpliSeq pipeline or VSEARCH (version 2.15.1; Rognes et al., 2016) depending on the OTU generation method used as described below.

#### 2.4.1 OTU generation

Soil eukaryote community composition was estimated by generating OTUs from the filtered reads using three different algorithms. In this study, we use the term OTU_C to refer to the output of such centroid based method, OTU_S for the output of such single‐linkage based clustering method and for consistency, instead of ASV we use OTU_A to refer to the output of this denoising method. The selected algorithms have different principles for OTU generation and are all commonly used in metabarcoding studies. The OTU_A dataset consisted of ASVs inferred using DADA2 in the AmpliSeq pipeline (Straub et al., 2020). This method is designed to identify true sequence variants in the amplicon library by collapsing variations derived from sequencing errors across all samples using the pooled function. The OTU_C dataset was generated by abundance‐based greedy clustering in VSEARCH (Rognes et al., 2016) with a similarity threshold of 99%. Finally, the OTU_S dataset was generated using single‐linkage “swarm” clustering with a distance threshold of 30 bp (approximately 2%) in GeFaST (Müller & Nebel, 2018). This threshold was selected to ensure that two copies of the same biological sequence, each containing a maximum of 15 different errors (i.e., 1% error in 1500 bp), would still be clustered together even if the error‐free seed sequence was absent. For OTU_C (VSEARCH) and OTU_S (GeFaST), the CCS reads corresponding to each OTU were extracted using a custom BASH script (Data S1), and a consensus sequence for each OTU was calculated using PacBio's c3s (consensus of CCS; https://github.com/PacificBiosciences/c3s), which calculates a consensus sequence using SPOA (Vaser et al., 2017) with base quality scores used as weights. This way the sequences representing all three types of OTUs were inferred with a quality‐aware method. Chimeric sequences were removed from all datasets using the removeBimeraDenovo function of DADA2. Global singletons (which are not generated by DADA2) were also removed from the OTU_C and OTU_S datasets before further analysis.

### 2.5 Placing OTU sequences in a phylogenetic and taxonomic context

#### 2.5.1 Taxonomy assignment and maximum likelihood (ML) tree based on the 5.8S and LSU region

The LSU, 5.8S, ITS2, and full ITS (ITS1–5.8S–ITS2) regions were extracted using LSUx (version 0.99.6; https://github.com/brendanf/LSUx; Furneaux et al., 2021) from the OTU consensus sequences generated by all three OTU generation methods. Three datasets were used for assigning taxonomy: the SILVA LSU NR 99 dataset (version 138.1, eukaryotes only; Quast et al., 2012), RDP fungal LSU training set (version 11; Liu et al., 2012) for the extracted LSU sequences, and the UNITE all‐eukaryotes dataset (version 8.2, including singletons; (Nilsson et al., 2019) for the extracted ITS sequences. The taxonomic annotations for all three reference datasets were mapped to the UNITE classification system so that assignments from different datasets could be compared (reUnite version 0.2.0; https://github.com/brendanf/reUnite; Furneaux et al., 2021). Taxonomy was assigned using the SINTAX algorithm (Edgar, 2016) as implemented in VSEARCH (version 2.15.1; Rognes et al., 2016) with a bootstrap threshold of 0.8.

Unique 5.8S and LSU sequences from the combined (OTU_A, OTU_C, OTU_S) dataset were independently aligned with DECIPHER (version 2.18.0; Wright, 2015). The LSU alignment was truncated at a position corresponding to 879 in the S288C reference sequence due to the presence of introns after this position. The 5.8S and LSU alignments were then concatenated, and each sequence in the concatenated alignment was assigned a unique identifier based on its component 5.8S and LSU sequences. A preliminary ML phylogenetic tree was generated from the concatenated alignment using fasttree (version 2.1.10; Price et al., 2010) with the GTR+C model with 20 rate categories. For sequences assigned at the kingdom level without conflicts between reference databases, the ML tree search was constrained by requiring that each kingdom form a monophyletic clade. Monophyly of the eukaryotic supergroups found in the samples was also constrained (Figure S1) according to the current consensus of phylogenomic studies (Adl et al., 2019; Strassert et al., 2019). The position of sequences which were not identified to kingdom, or which received conflicting kingdom assignments from the different reference datasets, was not constrained. The tree was rooted with sequences representing the protist phyla Discoba (Data S2).

The clades corresponding to animals (kingdom Metazoa, 90 OTUs) and vascular plants (phylum Streptophyta, 42 OTUs) were identified from the tree, and OTUs corresponding to those sequences were removed from further analyses. In addition, the clade corresponding to kingdom Fungi was extracted and analyzed separately from protists. For kingdom Fungi only, a refined alignment and phylogenetic tree were generated by realignment of the 5.8S and LSU regions using MAFFT‐ginsi (Katoh & Standley, 2016), including truncation of the LSU alignment as above, followed by ML phylogeny construction using IQ‐TREE (Nguyen et al., 2015; Stamatakis, 2014) using the built‐in ModelFinder Plus (Kalyaanamoorthy et al., 2017), which selected the TIM3+F‐R10 model, and 1000 ultrafast bootstrap replicates (Hoang et al., 2018). The most abundant Holozoan OTU (across OTU_A, OTU_C, and OTU_S) from the dataset (an Ichthyosporian) was retained to root the fungal tree (Data S3).

#### 2.5.2 Comparing different types of OTUs clustering methods

To analyze detection limits and taxonomic resolution of the three methods used to infer OTUs, we plotted the Fungi‐only tree along with a heatmap of the average relative read abundance across all samples in separate columns for OTU_A, OTU_C and OTU_S (Data S3). To explore the phylogenetic resolution of the three methods, the ITS2 regions extracted from each sequence by LSUx (as described above) were clustered using the same methodology outlined in Kõljalg et al. (2013) to generate UNITE SH at 97 and 99% sequence similarity: sequences were first pre‐clustered at 80% sequence similarity by VSEARCH, and then the sequences within each precluster were clustered at 97% and 99% similarity by BLASTCLUST (version 2.2.26; Altschul et al., 1990; Dondoshansky & Wolf, 2000). In addition to these, respectively, lax and more stringent species‐level thresholds, we also generated approximately genus‐level clusters using a 90% (GH_90) similarity threshold (Tedersoo et al., 2014). We then mapped the three ITS2 clustering levels onto the phylogenetic tree to determine how many clusters were monophyletic and how well the three different OTU generation methods captured diversity at different threshold levels. Further, we estimated the abundance necessary for a taxon to be detected as an OTU_A. For this, we used the average read abundance of OTU_S and OTU_C sequences assigned to GH_90, SH_97 and SH_99 ITS2 clusters to identify the detection limit of DADA2.

#### 2.5.3 OTU accumulation curves

OTU accumulation curves and asymptotic species richness estimates were calculated using the iNEXT package (Hsieh et al., 2016) in R (version 4.0.4; R Core Team, 2019). For this analysis, accumulation curves for OTUs, SH_99, SH_97 or GH_90 were calculated separately for the three clustering methods, the two different soil moisture regimes (wet and mesic‐dry) based on the number of raw reads obtained in each soil moisture regime and the number of samples.

### 2.6 Statistical analysis

#### 2.6.1 Plant and soil microeukaryotes community analysis

Statistical analysis were performed in R using RStudio (RStudio Team, 2015). The community composition of plant species recorded in all plots along transects 1 and 4 was visualized by nonmetric Multidimensional Scaling (nMDS) using Bray‐Curtis dissimilarities. Both plant and belowground analyses were made with the vegan software package (version 2.5‐7; Oksanen et al., 2019). The two‐sample I‐test was used to test for significant difference in mean gravimetric soil moisture between the two soil moisture regimes (wet vs. mesic‐dry). The three OTU occurrence tables were transformed to relative abundances for each sample and used for community analysis. nMDS ordination plots were generated using the “metaMDS”function in vegan. To down weight the importance of common taxa, the analysis was repeated using square root transformation of relative abundance data prior to calculation of the Bray‐Curtis dissimilarity. Marginal and individual PERMANOVAs were conducted on all three datasets and two standardizations described above, using the “adonis” function in the vegan to test for the marginal and overall effect of soil moisture regime (wet vs. mesic‐dry) and presence/absence of *F*. *meleagris* on shaping belowground microeukaryotic communities at the study site.

After taxonomic assignment as described above, the three OTU occurrence tables were divided into two separate datasets for fungal and protist communities separately (i.e., nonfungal microeukaryotes). The ordination and PERMANOVA tests described above were repeated for these taxonomically distinct communities.

#### 2.6.2 Using OTU_S to explore community composition in different conditions

Distribution and abundance of OTU_Ss were visualized across the contrasting soil conditions wet versus mesic‐dry soil conditions and presence/absence of *F*. *meleagris* using a Venn‐diagram (Heberle et al., 2015). The relative abundance of unique and shared fungal and protist OTU_Ss were calculated across samples for the two contrasting conditions. To identify differentially abundant taxa in contrasting soil moisture regimes (wet vs. mesic‐dry) and presence/absence of *F*. *meleagris*, the “phyloseq‐to‐deseq” function in the phyloseq package (v 1.34.0; McMurdie & Holmes, 2013) was applied to OTU_S occurrence tables separately. The generated phyloseq object was analyzed using the DESeq2 tool (DESeq package version 1.30.1; Love et al., 2014). Identified taxa and their differential abundance were illustrated using the ggplot2 R package (Wickham, 2016).

## 3 RESULTS

### 3.1 Plant community shift across sharp soil moisture transition at the Kungsängen meadow

**FIGURE 2:**
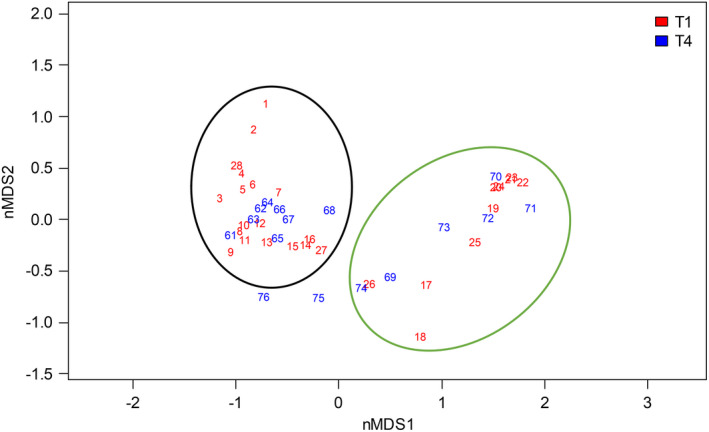
Non‐metric multidimensional scaling (nMDS) ordination of the plant community in plots along transect T1 (plots 1–28, red) and T4 (plots 61–76 blue). Ellipses outline the distribution of plots from the wet part (green) and mesic‐dry part (black). Plots 75 and 76 are on the levee close to the river with somewhat deviating vegetation. The closest plots to the locations for soil microbiome sampling (enclosed by dashed ellipses) on the mesic‐dry side were plots 14–16 (T1) and 67–68 (T4) and on the wet side plots 17–18 (T1) and 69 (T4)

The plant community was assessed in plots along two permanent transects stretching from the wetter part close to the Fyris River to a mesic‐dry part of the meadow (Figure 1). A total of 85 plant species were recorded along transects 1 and 4 (73 and 61 species, respectively; Data S4), with the highest numbers, 24–29 plant species, recorded in plots 13–16 in transect 1 (Figure S2), just on the mesic‐dry side of the moisture transition. In accordance with earlier inventories (Sernander, 1948; Zhang, 1983), the number of recorded plant species dropped rapidly in the wet part of the meadow with on average only six species per plot across plots 17–25. There is a slight levee along the river where the number of recorded species increases again (Figure S2). nMDS ordination of the plant community along transects 1 and 4 demonstrates the distinct separation between plots in the mesic‐dry part east of the soil sampling compared with the wet part west of the soil sampling (Figure 2), and with a transition from *Carex disticha* to *Carex acuta* dominance at the border (Zhang, 1983). *Alopecurus pratensis* and *Stellaria graminea* were detected in all plots in the mesic‐dry area and *Poa trivialis*, *Phleum pratense*, and *Trifolium repens* were other highly abundant species in the mesic‐dry area (Data S4). *Fritillaria meleagris* was frequent in plots in the mesic‐dry part to the east of the soil sampling (and also in elevated plots closest to the river) but did not occur in the wetter parts of the transects (Figure S2). The distinct *C*. *acuta* dominated community in the wet side of the meadow has been previously reported (Zhang, 1983). Other frequently observed species in the wet area include *Equisetum fluviatile*, *C*. *disticha*, and *Galium palustre*. Soil sampling in early June confirmed that mean gravimetric soil moisture was significantly different (*p *< .001; *t* = −5.1812, *n*_wet_ = 10; *n*_dry_ = 10) on either side of the plant community transition border, with 76% and 34% soil moisture in the wet compared with the mesic‐dry side of the meadow (Figure S3).

### 3.2 Characterizing the belowground microeukaryotic community composition

#### 3.2.1 Delimitation and identification of microeukaryotic OTUs

The three different OTU generation methods infer somewhat different community compositions from sequenced long‐read rDNA environmental DNA amplicons. For instance, the methods resulted in very different estimates of total non‐singleton OTU richness, ranging from 1,336 OTU_A detected based on inference of ASVs, compared with 2,046 OTU_S and 2,488 OTU_C for sequence similarity‐based clustering using single‐linkage or centroid‐based clusters, respectively (Table 1). The OTU_A dataset represents only 28% of the raw reads while the two other methods were comparable, capturing 81–83% of the reads into OTUs (Table 1). After pooling all reads, OTU accumulation curves for the three methods indicate that sequencing depth was sufficient to reach comparable asymptotic OTU richness estimates in both mesic‐dry and wet soil conditions (Figure S4a). For individual samples, however, increased sequencing depth would be expected to increase OTU detection for OTU_S and OTU_C but not for OTU_A (Figure S5). Further, the estimated asymptotic OTU richness increased for all methods when analyzing the pooled reads based on number of samples (Figure S4b), indicating that taking additional samples would be expected to increase the number of detected taxa for all methods. Across the three methods, 38–42% of the detected OTUs were taxonomically assigned to kingdom Fungi. Based on read abundance, the estimated proportion of fungi ranged from 34% for OTU_As compared with just over 40% for the two other methods (Table 1). Protists thus dominated the sequenced microeukaryotic soil community at this site.

**TABLE 1:** Number of inferred OTUs and the number of reads represented, for total microeukaryotic community and (fungi), for the three different clustering methods

Inferred OTUs	Total OTUs (Reads)	Out of total reads (%)	Fungal OUT (Reads)	Protists OTU (Reads)
OTU_A	1336 (14,056)	28.4	554 (4784)	662 (7238)
OTU_C	2488 (41,380)	81.3	933 (16,541)	1,168 (20,195)
OTU_S	2046 (42,353)	83.4	769 (17,130)	925 (20,653)

#### 3.2.2 OTU generation methods captured consistent community patterns across soil conditions

**FIGURE 3:**
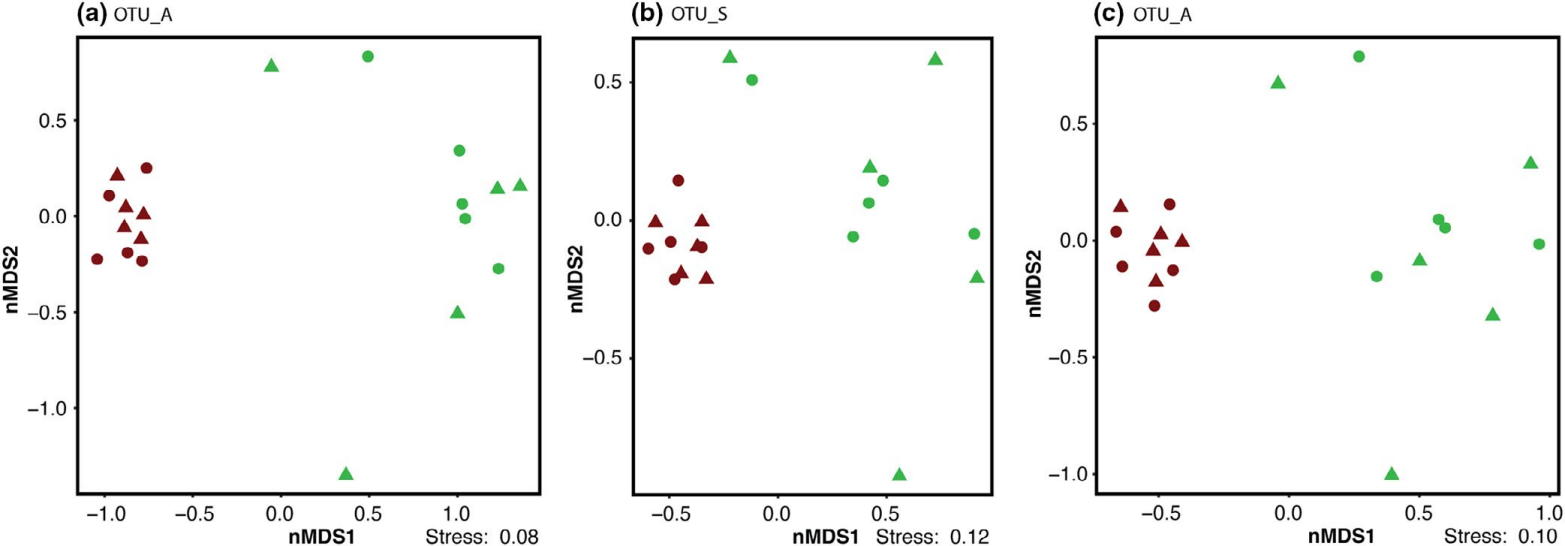
Non‐metric multidimensional scaling (nMDS) ordination of microeukaryotic communities recovered from wet (green) and mesic‐dry (brown) soil moisture regimes at the Kungsängen Nature Reserve using Bray‐Curtis dissimilarities calculated from relative abundance based on three different OTU inference methods (a) OTU_A, (b) OTU_S, and (c) OTU_C. Circles are samples with a *F*. *meleagris* plant and triangles are samples without *F*. *meleagris* plant

All three OTU generation methods consistently demonstrate that total soil microeukaryotic community composition clearly differentiated based on soil condition (wet or mesic‐dry), but no pattern was detected in relation to the presence of *F*. *meleagris*, as observable in nMDS ordinations (Figure 3). The observed separations were statistically significant (*p *= .001) as indicated by a marginal PERMANOVA test (Table S4) and remained when the importance of rare OTUs was down‐weighted by square root transformation of relative abundances (Figure S6, Table S5). Similar to observations for the plant community (Figure 2), microeukaryotic community composition was more variable among samples in wet soil conditions compared with mesic‐dry soil conditions (Figure 3). Mesic‐dry samples clustered closer together indicating that communities were more similar across samples (Figure 3). When analyzing fungal and protist community composition separately, we observed the same significant separation (*p *< .001) based on soil conditions (wet or mesic‐dry), but not in relation to the presence of *F*. *meleagris* (Figure S7, Table S6). While still significant, the separation is visually less distinct for the protist community based on OTU_S and OTU_C (Figure S7d,f) compared with OTU_A (Figure S7b). The tight clustering of samples from mesic‐dry conditions is recovered in both fungal and protist communities (Figure S7).

**FIGURE 4:**
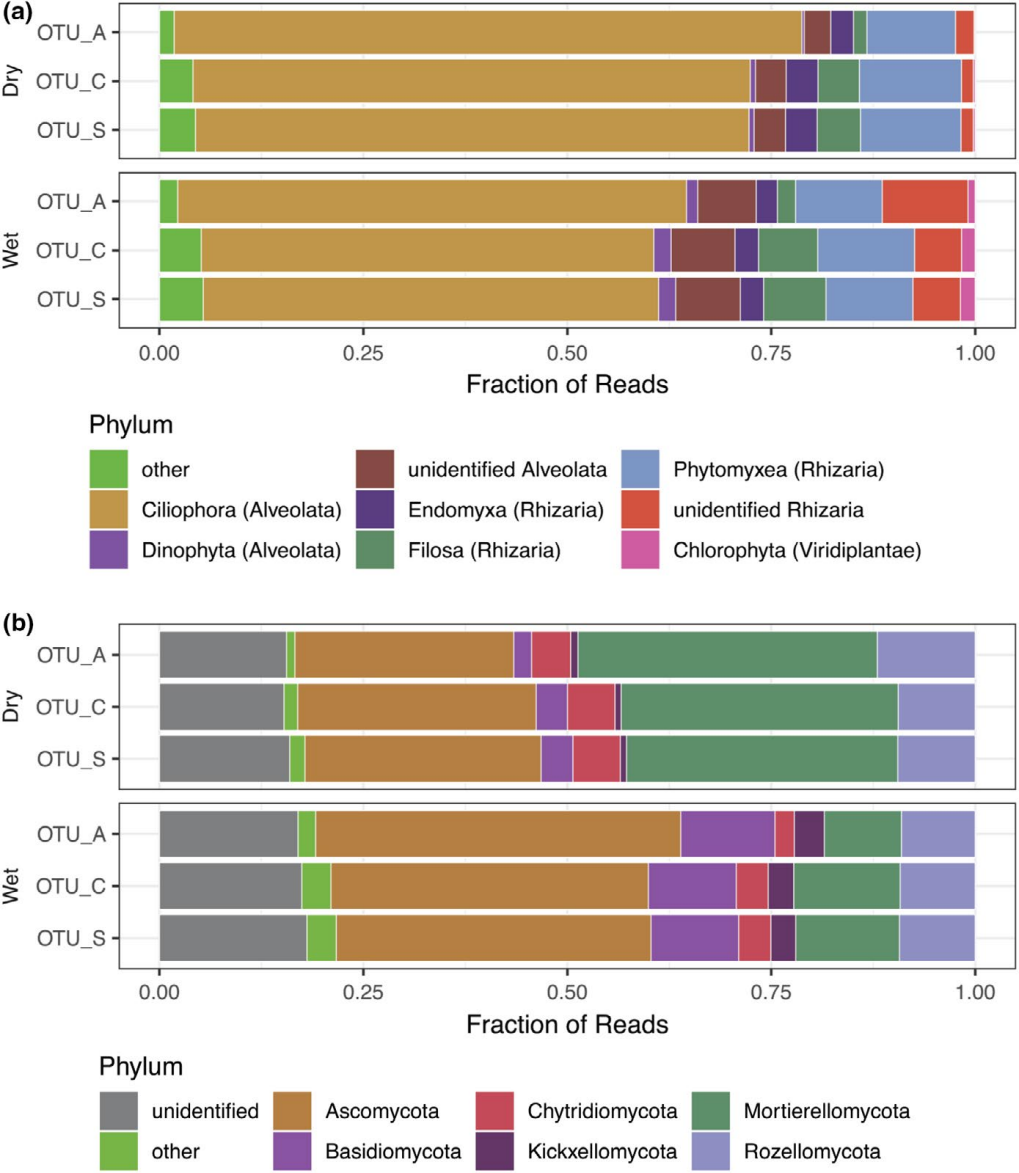
Phylum‐level taxonomic assignments of microeukaryotic communities in wet and mesic‐dry soil moisture regimes separated into (a) protists and (b) Fungi. Illustrated as mean fractional read abundance for the three occurrence tables OTU_A, OTU_C, and OTU_S. Phyla which represent <1.5% of total reads are grouped together as “other”

Overall, phylum‐level taxonomic composition was also comparable across the three OTU generation methods used (Figure 4). Based on read abundance, protist communities were dominated by the Ciliophora (Alveolata) in both wet and mesic‐dry soil conditions (Figure 4a). The relative abundance of Alveolata was slightly lower when communities were characterized using OTU_C and OTU_S compared with the OTU_A dataset (Figure S8). The Rhizarian phyla Endomyxa, Phytomyxea, and Filosa were also observed in both conditions and were more abundant when reads were clustered into OTU_C and OTU_S compared with OTU_As (Figure 4a). In wet conditions, a larger proportion of reads within both Alveolata and Rhizaria could not be identified at the phylum level highlighting the potential for future studies of poorly known lineages at this site. The proportion of Ciliophora was smaller in wet vs. mesic‐dry conditions. In both soil conditions, Ascomycota was the most common fungal phylum, and together with Basidiomycota, made up over half of the sequenced fungal community in wet soil conditions (Figure 4b). In mesic‐dry soil conditions on the other hand, Mortierellomycota made up a larger fraction of the reads, around 30%. Sequences assigned to Glomeromycota, which encompass all arbuscular mycorrhizal fungi, were rare at this site, despite known high abundance based on spore counts (personal observations of S.E.S.). Chytridiomycota were also more abundant in mesic‐dry compared with wet soil conditions, while Rozellomycota made up around 10% of the reads in both conditions. Close to 20% of fungal OTUs remained unidentified at phylum level across all three methods (Figure 4b). Many of these unidentified lineages cluster with Zoopagomycota, Kickxellomycota, and Rozellomycota in the fungal tree (Data S3).

### 3.3 Different OTU generation methods strongly influence species richness estimates

Overall community composition is captured well across the three OTU generation methods when analyzing ecological patterns (Figure 3) and relative abundance at the phylum level (Figure 4). However, the OTU generation methods differentially capture and represent the taxa in these communities, so that from the same raw reads, different sequences are selected to represent the clustered raw reads in the three datasets. The dependence on abundant seed sequences for denoising resulted in fewer OTU_As compared with the two other methods and entire lineages of rare taxa remained undetected with this method, while a large number of OTU_As are recovered from abundant taxa such as Mortierellomycota (Figure 4b and Figure S9). The detection limits of different OTU generation methods were compared by generating approximately genus‐level clusters using sequence similarity thresholds at 90% and species‐level clusters at either 99 or 97% across the ITS2 region extracted from all OTU representative sequences. Only 36% of all genus‐level clusters (GH_90) in the dataset were represented by an OTU_A sequence, compared with 94 and 96% for OTU_C and OTU_S, respectively (Table 2). The level of detection for SHs represented by up to 50 reads was lower for OTU_A than the other methods. In some cases, even close to 300 reads was not enough to detect a SH_99 with OTU_A (Figure S10). Even the more inclusive methods did not capture exactly the same genus‐level diversity, with just over 7% of all GH_90 represented by a sequence recovered by a single method (Table 2). However, no GH_90 was represented only by an OTU_A sequence.

**TABLE 2:** OTU sequences were clustered across the ITS2 region to represent taxa at different taxonomic ranks genus (GH_90) and species (SH_97 and SH_99), number of clusters, and % of these including sequences from the three OTU inference methods

Taxonomic rank (% ITS2 similarity)	Total	OTU_A (%)	OTU_C (%)	OTU_S (%)	Only one method
GH_90	1590	36	95	97	120
SH_97	2000	37	95	90	234
SH_99	2356	37	92	79	482

**FIGURE 5:**
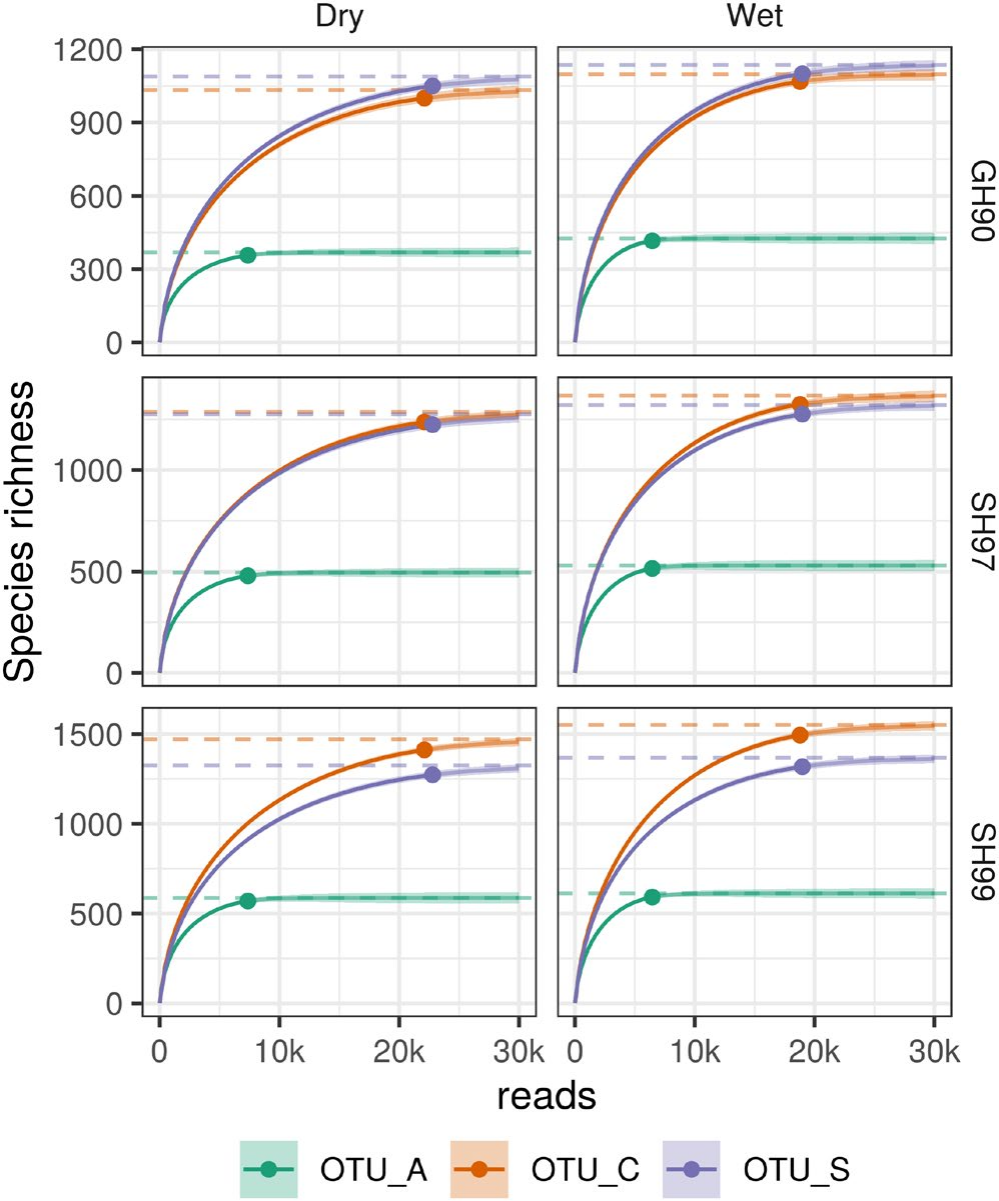
Species richness curve estimated from the bottom up as SH_99, SH_97, and GH_90, based on all reads combined for ten samples each from mesic‐dry and wet soil moisture regimes. OTUs were inferred using three different clustering methods: OTU_A (green), OTU_C (orange), and OTU_S (purple) and the ITS2 region of their representative sequences were then clustered into species and genus hypotheses (SH and GH), using three different ITS2 sequence similarity thresholds 99% for SH_99, 97% for SH_97, and 90% for GH_90

Species richness estimates are heavily influenced by the OTU generation method used with the lowest numbers estimated with OTU_A for all three ITS2 sequence similarity levels GH_90, SH_97 and SH_90 (Figure 5). While OTU_A richness was estimated to saturate close to 1000 in both wet and mesic‐dry soil conditions (Figure S4), these may represent only half as many species since the intraspecies variation is collapsed to around 600 SH_99 and just over 500 SH_97 (Figure 5). OTU richness estimates are highest for OTU_C at almost 1,700 followed by OTU_S at almost 1400 (Figure S4), and the numbers are only slightly lower when estimating species richness as SH_99 (Figure 5). Accepting ITS2 sequence similarity at either 99 or 97% as a proxy for species suggests that clustering into OTU_C or OTU_S detects close to three times as many species compared with denoising into OTU_A. Of the three methods, OTU_S is also the method that has the largest number of SH_99 and SH_97 represented by only one OTU (Figure S11) suggesting that in the long‐reads in the current dataset the OTU_S method provides the best estimate of species richness, as estimated by the SH_99 clusters of ITS2 regions in all representative sequences.

#### 3.3.1 Phylogenetic resolution of different OTU generation methods within kingdom Fungi

For a more detailed analysis of kingdom Fungi, phylogenetic reconstruction using the LSU and 5.8S regions of all fungal OTU representative sequences from the three OTU generation methods (Table 1) was used to analyze the phylogenetic signal of estimated species richness for the three different OTU generation methods (Data S3). Within kingdom Fungi, we identified 1,590 genus‐level clusters (GH_90), the vast majority of which were monophyletic, indicating good concordance between phylogenetic inference based on conserved LSU and 5.8 regions and sequence similarity in the ITS2 region of individual sequences. The nine GH_90 clusters that were polyphyletic in the fungal tree were found in lineages with short branch lengths separating terminal nodes (Figure S12a). In these lineages, the existing variation within conserved regions, which may be in part due to sequencing errors, provides low phylogenetic resolution, resulting in collapse to random order in the tree. The same pattern applies to cases of polyphyletic SH_97 and SH_99 clusters since different sequence regions were used for phylogenetic inference and similarity clustering (Data S3).

As expected, different OTU generation methods detect different levels of genetic variation within and between taxa in the sequenced fungal community. For rare taxa, OTU_A completely fails to even detect phylum level diversity, as in the case of Glomeromycota, that was recovered in six OTUs across OTU_C and OTU_S, all of which represent rare taxa in the dataset (Figure S12b). In abundant taxa on the other hand, intra‐species variation is captured with several OTU_A sequences per SH_99 or SH_97 (Figure S10), while the other methods identify one or two variants as exemplified by a single *Morteriella* SH_99, containing 12 OTU_As (Figure S12c).

### 3.4 Differentially abundant taxa in microeukaryote community

Based on the consistency between number of OTU_S and SH_99, we conclude that the OTU_S dataset provides a better estimate of total species richness. We thus used this dataset for further analysis of differences in communities associated with contrasting soil conditions. Across the total microbial eukaryotic community, 282 fungal and 383 protist OTU_Ss were present in both wet and mesic‐dry conditions. A total of 195 fungal and 243 protists OTU_Ss were presented only in wet conditions, while 292 fungal and 299 protists OTU_Ss were detected only in mesic‐dry condition (Figure S13). Based on the DESeq analysis, only 15 were significantly differentially abundant across all OTU_S (Figure S14). Four out of fifteen were protists, one of them belongs to genus *Polymyxa* and is only found in mesic‐dry condition while the other three are found predominantly in wet soil condition (Data S5). Taxa in at least two of the *Polymyxa* genus have been reported as plant root endoparasites (Decroës et al., 2019; Neuhauser et al., 2014). Of the eleven OTU_S belonging to the fungal kingdom, only one was significantly more abundant in the mesic‐dry condition. Five OTU_Ss belong to Ascomycota were identified until genus level (three *Cistella*, *Psedeurotium*, and *Stagonospora*), and two OTU_Ss belong to Basidiomycota, recognized to the order level. All these taxa were significantly more abundant in the wet condition (Data S5). Detection of significant association with the contrasting soil conditions is limited by the current sampling design with only ten samples from wet and mesic‐dry, respectively. Additional samples would have captured more of the local community. In relation to the presence/absence of *F*. *meleagris*, 160 fungal and 231 protists OTU_Ss were detected only in *F*. *meleagris* samples, while 144 fungal and 159 protist OTU_Ss were observed in samples without *F. meleagris*. In total, 465 fungal and 544 protist OTU_Ss were observed in both with and without *F. meleagris* samples (Figure S15). Taking into account the low number of samples no OTU_Ss were differentially abundant based on the DESeq analysis. This is likely a result of sampling large soil volumes with multiple microhabitats, where only some are affected by the target plant species. Our attempt to also sequence root associated communities, which would be expected to better capture specifically host plant associated microorganisms, failed due to low success rate of microeukaryote amplification from *F*. *meleagris* root samples (data not shown).

## 4 DISCUSSION

In this study, we used a distinct transition zone in vegetation and soil moisture, as the framework to analyze how different OTU generation methods affect the detection of a shift in the composition of microeukaryotic soil communities. Interestingly, the sharp transition in plant community, with lower richness in wet compared with mesic‐dry soils, was not associated with a difference in observed richness for the corresponding microeukaryotic soil communities. However, both plant and soil microeukaryote community compositions were significantly different in wet and mesic‐dry soil moisture regimes. We demonstrate that different OTU generation methods applied to the same long amplicon eDNA dataset affect the documented composition of soil microeukaryotic communities. Similarly, earlier studies have reported that large‐scale ecological patterns are recovered irrespective of the OTU or ASV generation method or clustering threshold (for short‐read data) (Glassman & Martiny, 2018), sequencing technology (Furneaux et al., 2021) or sampling effort (Castle et al., 2019). We conclude that large‐scale ecological patterns are robustly recovered irrespective of the OTU or ASV generation method applied. However, for studies focused on the particular members of these contrasting communities, the OTU generation method selected significantly affects the phylogenetic resolution and detection of taxa. For instance, our results show that inference of ASVs with DADA2 (here OTU_A) captures less than 30% of all reads, providing information on intra‐species genetic variation only for abundant taxa while rare taxa, including entire phylum‐level lineages, remain undetected. The overall estimated OTU richness was also lower for OTU_A compared with the cluster‐based methods. When comparing OTU generation methods, others have found contrasting patterns, with higher richness captured with ASVs, the equivalent to OTU_A in this study, compared with clustering (Glassman & Martiny, 2018). Differences between our results and those of Glassman and Martiny (2018) can be attributed to the earlier study's shorter amplicon (only ITS2 for fungi) and higher sequencing depth generated by Illumina, compared with our long amplicon sequencing with lower depth using PacBio. In studies using short‐read amplicons, denoising increased overall richness by capturing intraspecies genetic variation (Callahan et al., 2016). However, when applied to long‐read amplicons from diverse communities, intra‐species variation can only be captured for the most abundant taxa. While OTU accumulation curves saturated for all methods, we found that increasing the number of samples would have increased the number of detected taxa at the site. Due to soil heterogeneity and spatial community turnover, increasing the number of samples rather than the sequencing depth increases the estimated alpha diversity even in well‐mixed, managed agricultural soils (Castle et al., 2019). The same pattern was previously observed in forest soils from West Africa (Meidl et al., 2021), highlighting the importance of optimizing sampling effort versus sequencing depth to obtain a good representation of the alpha diversity.

Single‐linkage clustering with a distance threshold of 2%, on the other hand, captures most reads in OTU_Ss that correspond closely to broadly accepted fungal species‐level sequence similarity across the ITS region, suggesting that this method provides an acceptable proxy for species richness. We anticipate that phylogenetic resolution of species‐ and genus‐level relationships could have been improved by the generation of a hybrid tree that included ITS2 alignments to resolve relationships within each GH_90 lineage, in a manner similar to ghost‐tree (Fouquier et al., 2016). Such tree could have been used to generate phylogenetic SH (ref) to analyze community composition and generate species richness estimates for these communities. Apart from sequence clustering approaches, extraction and amplification biases remains as a major filtering step for analysis of total microeukaryotic soil communities. Although the primers we used have no known biases against Glomeromycota, we obtained low read abundance for this group, despite known high abundance of Glomeromycota spores at the site. This apparent contradiction may be explained by the low copy number of around ten rDNA operones in this phylum (Maeda et al., 2018) compared with other fungi that may harbor hundreds to thousands of copies (Lofgren et al., 2019). In addition to copy number variation, length difference in the rDNA, especially ITS, can introduce bias both during PCR and sequencing (Leho Tedersoo et al., 2015), rendering this type of data far from quantitative, especially when applied to broad phylogenetically groups such as microeukaryotes.

Our study also provides a first insight into the belowground diversity of microeukaryotes in a meadow known for its rich plant community (Sernander, 1948; Zhang, 1983; Zhang & Hytteborn, 1985). Studies that aim to simultaneously characterize communities of both protists and fungi have often found that fungi dominate the sequenced microeukaryotic communities, for example, in tropical forest soil (Tedersoo et al., 2018) and soils from different habitats in temperate regions (Tedersoo & Anslan, 2019). In previous studies using the exact same primers, sequenced soil communities from ectomycorrhizal dominated forests in Sweden and West Africa have been almost completely dominated by reads taxonomically assigned to kingdom Fungi (Furneaux et al., 2021; Kalsoom Khan et al., 2020; Meidl et al., 2021). The dominance of protists in the sequenced microeukaryotic community indicates that these soil systems are particularly suitable for diverse communities of protists. High soil moisture may be one explanation, but other factors like plant community, pH and total nitrogen have also been associated with high abundance of protists in soil (Oliverio et al., 2020). We anticipate that future studies may hold many interesting discoveries of hitherto unknown diversity at this site.

## CONFLICT OF INTEREST

The authors state no conflict of interest.

## AUTHOR CONTRIBUTION

**Shadi Eshghi Sahraei:** Data curation (equal); Formal analysis (equal); Validation (equal); Visualization (equal); Writing – original draft (equal). **Brendan Furneaux:** Conceptualization (equal); Data curation (equal); Formal analysis (lead); Methodology (lead); Validation (supporting); Visualization (equal); Writing – original draft (supporting). **Kerri Kluting:** Data curation (equal); Formal analysis (supporting); Writing – original draft (supporting). **Mustafa Zakieh:** Investigation (supporting); Methodology (supporting); Writing – original draft (supporting). **Håkan Rydin:** Investigation (supporting); Methodology (supporting); Writing – original draft (supporting). **Håkan Hytteborn:** Investigation (supporting); Methodology (supporting); Writing – original draft (supporting). **Anna Rosling:** Conceptualization (equal); Funding acquisition (lead); Investigation (equal); Methodology (equal); Project administration (lead); Resources (lead); Supervision (lead); Validation (equal); Writing – original draft (equal).

## Supporting information

Supplementary Material

Data S1

Data S2

Data S3

Data S4

Data S5

## Data Availability

Raw reads, accompanying meta‐data and representative OTU_S sequences are available in ENA under the accession no. PRJEB47280.
