# Supplementary material for: Effects of operational taxonomic unit inference methods on soil microeukaryote community analysis using long‐read metabarcoding
Source: Ecol Evol. 2022 Mar 8;12(3):e8676. doi: 10.1002/ece3.8676 (PMC8928899; doi:10.1002/ece3.8676)
Supplement: Supplementary file 1 — Supplementary Material [file ECE3-12-e8676-s005.docx]

**Supplementary tables and figures:**

**Table S1.** Common plant species at soil sampling

**Table S2.** Gravimetric soil moisture

**Table S3.** Sample name and primer tags

**Table S4.** Marginal PERMANOVA based on Bray – Curtis on relative abundance of total micro-eukaryotic community

**Table S5.** Marginal PERMANOVA based on Bray – Curtis on square root transformed relative abundance of total micro-eukaryotic community

**Table S6.** Marginal PERMANOVA based on Bray – Curtis on relative abundance of fungi and protists separately

**Figure S1.** Constraints for preliminary all-eukaryote ML tree

**Figure S2.** Number of plant species recorded in plots 1-28 on transect 1

**Figure S3.** Gravimetric soil moisture

**Figure S4.** OTU richness curves for dry and wet soil, based on combined reads and samples

**Figure S5.** OTU richness curves for dry and wet soil, based on samples separately

**Figure S6**. nMDS of soil micro-eukaryotic community based on square root transformed relative abundances of OTU_A, OTU_S and OTU_C

**Figure S7.** nMDS of soil fungi and protists separately based on relative abundances of OTU_A, OTU_S and OTU_C

**Figure S8.** Kingdom-level taxonomic assignment of protists as fraction of OTUs and reads

**Figure S9.** Phylum-level taxonomic assignment of fungi as fraction of OTUs

**Figure S10.** Number of OTU_As recovered per species hypothesis (SH)

**Figure S11.** Number of SH_99, SH_97 and GH_90 clusters represented by different number of OTUs

**Figure S12.** Phylogenetic sister lineages with sequences from the three OTU methods

**Figure S13.** Venn diagram and relative abundance of shared and unique OTU_S across wet and mesic-dry soil conditions

**Figure S14.** Differentially abundant OTU_Ss between wet and mesic-dry soil conditions

**Figure S15.** Venn diagram and relative abundance of shared and unique OTU_S across samples with and without Fritillaria

**Data S1.** BASH Script for consensus sequences from CCS reads

**Data S2.** Tree with all micro-eukaryotic sequences from the site

**Data S3.** Tree with all fungal sequences from the site

**Data S4.** Plant species along transects 1 and 4

**Data S5.** Significantly different OTU_S

**Table S1.** Common plant species collected and identified at the soil sampling locations, check marks indicate presence at the wet and Mesic-dry side of the soil moisture transition border

| **Plant taxonomy** | **Mesic-dry** | **Wet** |
| --- | --- | --- |
| *Alchemilla monticola* Opiz. | ✔ |  |
| *Alopecurus pratensis* L. | ✔ |  |
| *Carex acuta* L. | ✔ | ✔ |
| *Carex cespitosa* L. | ✔ |  |
| *Carex disticha* Huds.* | ✔ |  |
| *Caltha palustris* L. |  | ✔ |
| *Deschampsia cespitosa* L. | ✔ |  |
| *Equisetum fluviatile* L. |  | ✔ |
| *Fritillaria meleagris* L. | ✔ | ✔ |
| *Filipendula ulmaria* (L.) Maxim | ✔ | ✔ |
| *Geum rivale* L. | ✔ |  |
| *Lathyrus pratensis* L. | ✔ |  |
| *Poa trivialis* L. | ✔ |  |
| *Ranunculus acris* L. | ✔ |  |
| *Ranunculus auricomus* L. | ✔ |  |
| *Festuca pratensis* Huds. | ✔ |  |
| *Trifolium pratense* L. | ✔ |  |
| *Trifolium repens* L. | ✔ |  |

*One observation in the wet sample plots.

**Table S2.** Wet weight, dry weight, water weight and gravimetric soil moisture for soil samples collected from five locations in the east (E) and five locations in the west (W) side of the soil moisture transition zone at Kungsängen nature preserve. From each location two soil samples were collected, one around a *Fritillaria meleagris* plant (F) and one at least 0,5m away from a *Fritillaria meleagris* plant (N).

| Samples | Soil condition | Wet weight (g) | Dry weight (g) | Water weight (g) | Gravimetric soil moisture (%) |
| --- | --- | --- | --- | --- | --- |
| 1EFS | Mesic-dry | 4,07 | 2,95 | 1,12 | 38,0 |
| 2EFS | Mesic-dry | 6,07 | 4,49 | 1,58 | 35,2 |
| 3EFS | Mesic-dry | 6,57 | 5,11 | 1,46 | 28,6 |
| 4EFS | Mesic-dry | 4,26 | 2,76 | 1,5 | 54,4 |
| 5EFS | Mesic-dry | 3,87 | 3,31 | 0,56 | 17,0 |
| 1ENS | Mesic-dry | 5,85 | 3,86 | 1,99 | 51,6 |
| 2ENS | Mesic-dry | 3,09 | 2,48 | 0,61 | 24,6 |
| 3ENS | Mesic-dry | 4,78 | 3,85 | 0,93 | 24,2 |
| 4ENS | Mesic-dry | 6,16 | 4,26 | 1,9 | 44,6 |
| 5ENS | Mesic-dry | 4,87 | 4,13 | 0,74 | 17,9 |
| 1WFS | Wet | 3,24 | 2,33 | 0,91 | 39,1 |
| 2WFS | Wet | 8,37 | 4,47 | 3,9 | 87,3 |
| 3WFS | Wet | 5,68 | 3,06 | 2,62 | 85,6 |
| 4WFS | Wet | 5,07 | 3,09 | 1,98 | 64,1 |
| 5WFS | Wet | 6,45 | 3,42 | 3,03 | 88,6 |
| 1WNS | Wet | 6,03 | 3,3 | 2,73 | 82,7 |
| 2WNS | Wet | 7,02 | 3,77 | 3,25 | 86,2 |
| 3WNS | Wet | 8,37 | 3,9 | 4,47 | 114,6 |
| 4WNS | Wet | 7,77 | 5,26 | 2,51 | 47,7 |
| 5WNS | Wet | 4,87 | 2,95 | 1,92 | 65,1 |

**Table S3.** Sample name and corresponding forward and reverse primers and tags

| Sample | pad | Forward Primer | Forward barcode | Reverse barcode | Reverse primer | pad |
| --- | --- | --- | --- | --- | --- | --- |
| 1EFS | GGTAG | TCCGTAGGTGAACCTGC | TCAGACGATGCGTCAT | ACACTGACGTCGCGAC | TCCTGAGGGAAACTTCG | GGTAG |
| 1ENS | GGTAG | TCCGTAGGTGAACCTGC | TCAGACGATGCGTCAT | CGTCTATATACGTATA | TCCTGAGGGAAACTTCG | GGTAG |
| 2EFS | GGTAG | TCCGTAGGTGAACCTGC | TCAGACGATGCGTCAT | ATAGAGACTCAGAGCT | TCCTGAGGGAAACTTCG | GGTAG |
| 2ENS | GGTAG | TCCGTAGGTGAACCTGC | TCAGACGATGCGTCAT | TAGATGCGAGAGTAGA | TCCTGAGGGAAACTTCG | GGTAG |
| 3EFS | GGTAG | TCCGTAGGTGAACCTGC | TCAGACGATGCGTCAT | CATAGCGACTATCGTG | TCCTGAGGGAAACTTCG | GGTAG |
| 3ENS | GGTAG | TCCGTAGGTGAACCTGC | TCAGACGATGCGTCAT | CATCACTACGCTAGAT | TCCTGAGGGAAACTTCG | GGTAG |
| 4EFS | GGTAG | TCCGTAGGTGAACCTGC | TCAGACGATGCGTCAT | CGCATCTGTGCATGCA | TCCTGAGGGAAACTTCG | GGTAG |
| 4ENS | GGTAG | TCCGTAGGTGAACCTGC | TCAGACGATGCGTCAT | TATGTGATCGTCTCTC | TCCTGAGGGAAACTTCG | GGTAG |
| 5EFS | GGTAG | TCCGTAGGTGAACCTGC | CTATACATGACTCTGC | ACACTGACGTCGCGAC | TCCTGAGGGAAACTTCG | GGTAG |
| 5ENS | GGTAG | TCCGTAGGTGAACCTGC | CTATACATGACTCTGC | CGTCTATATACGTATA | TCCTGAGGGAAACTTCG | GGTAG |
| 1WFS | GGTAG | TCCGTAGGTGAACCTGC | CTATACATGACTCTGC | ATAGAGACTCAGAGCT | TCCTGAGGGAAACTTCG | GGTAG |
| 1WNS | GGTAG | TCCGTAGGTGAACCTGC | CTATACATGACTCTGC | TAGATGCGAGAGTAGA | TCCTGAGGGAAACTTCG | GGTAG |
| 2WFS | GGTAG | TCCGTAGGTGAACCTGC | CTATACATGACTCTGC | CATAGCGACTATCGTG | TCCTGAGGGAAACTTCG | GGTAG |
| 2WNS | GGTAG | TCCGTAGGTGAACCTGC | CTATACATGACTCTGC | CATCACTACGCTAGAT | TCCTGAGGGAAACTTCG | GGTAG |
| 3WFS | GGTAG | TCCGTAGGTGAACCTGC | CTATACATGACTCTGC | CGCATCTGTGCATGCA | TCCTGAGGGAAACTTCG | GGTAG |
| 3WNS | GGTAG | TCCGTAGGTGAACCTGC | CTATACATGACTCTGC | TATGTGATCGTCTCTC | TCCTGAGGGAAACTTCG | GGTAG |
| 4WFS | GGTAG | TCCGTAGGTGAACCTGC | TACTAGAGTAGCACTC | ACACTGACGTCGCGAC | TCCTGAGGGAAACTTCG | GGTAG |
| 4WNS | GGTAG | TCCGTAGGTGAACCTGC | TACTAGAGTAGCACTC | CGTCTATATACGTATA | TCCTGAGGGAAACTTCG | GGTAG |
| 5WFS | GGTAG | TCCGTAGGTGAACCTGC | TACTAGAGTAGCACTC | ATAGAGACTCAGAGCT | TCCTGAGGGAAACTTCG | GGTAG |
| 5WNS | GGTAG | TCCGTAGGTGAACCTGC | TACTAGAGTAGCACTC | TAGATGCGAGAGTAGA | TCCTGAGGGAAACTTCG | GGTAG |

**Table S4.** Marginal and individual PERMANOVAs of relative explanatory power of soil condition (Wet vs. Mesic-dry) and *F. meleagris* (Presence/Absence), on sequenced micro-eukaryotic communities across 20 soil samples. Bray Curtis dissimilarity index was calculated from relative abundance data from three different OTU occurrence tables (a) OTU_A, (b) OTU_C and (c) OTU_S.

| **(a) OTU_A** | **DF** | **Sum of Squares** | ***R^2^*** | ***F*** | ***Pr (>F)*** |
| --- | --- | --- | --- | --- | --- |
| Soil conditions (Wet vs. Mesic-dry) | 1 | 1.6575 | 0.24323 | 5.7184 | 0.001*** |
| *F. meleagris* P/A | 1 | 0.2296 | 0.03369 | 0.7920 | 0.740 |
| Residual | 17 | 4.9276 | 0.72308 |  |  |
| Total | 19 | 6.8147 | 1 |  |  |

| **(b) OTU_C** | **DF** | **Sum of Squares** | ***R^2^*** | ***F*** | ***Pr (>F)*** |
| --- | --- | --- | --- | --- | --- |
| Soil conditions (Wet vs. Mesic-dry) | 1 | 1.2933 | 0.22977 | 5.3135 | 0.001*** |
| *F. meleagris* P/A | 1 | 0.1974 | 0.03508 | 0.8112 | 0.717 |
| Residual | 17 | 4.1377 | 0.73515 |  |  |
| Total | 19 | 5.6284 | 1 |  |  |
|  |  |  |  |  |  |
| **(c) OTU_S** | **DF** | **Sum of Squares** | ***R^2^*** | ***F*** | ***Pr (>F)*** |
| Soil conditions (Wet vs. Mesic-dry) | 1 | 1.0629 | 0.21489 | 4.8796 | 0.001*** |
| *F. meleagris* P/A | 1 | 0.1803 | 0.03645 | 0.8278 | 0.694 |
| Residual | 17 | 3.7031 | 0.74866 |  |  |
| Total | 19 | 4.9463 | 1 |  |  |

**Table S5.** Marginal and individual PERMANOVAs of relative explanatory power of soil condition (Wet vs. Mesic-dry) and *F. meleagris* (Presence/Absence), on sequenced micro-eukaryotic communities across 20 soil samples. Bray-Curtis dissimilarity index was calculated from square root transformed relative abundance data from three different OTU occurrence tables (a) OTU_A, (b) OTU_C and (c) OTU_S.

| **(a) OTU_A** | **DF** | **Sum of Squares** | ***R^2^*** | ***F*** | ***Pr (>F)*** |
| --- | --- | --- | --- | --- | --- |
| Soil conditions (Wet vs. Mesic-dry) | 1 | 1.4492 | 0.22286 | 5.1248 | 0.001*** |
| *F. meleagris* P/A | 1 | 0.2462 | 0.03786 | 0.8706 | 0.577 |
| Residual | 17 | 4.8074 | 0.73928 |  |  |
| Total | 19 | 6.5028 | 1 |  |  |

| **(b) OTU_C** | **DF** | **Sum of Squares** | ***R^2^*** | ***F*** | ***Pr (>F)*** |
| --- | --- | --- | --- | --- | --- |
| Soil conditions (Wet vs. Mesic-dry) | 1 | 1.1754 | 0.21304 | 4.8358 | 0.001*** |
| *F. meleagris* P/A | 1 | 0.2099 | 0.03803 | 0.8633 | 0.624 |
| Residual | 17 | 4.1322 | 0.74893 |  |  |
| Total | 19 | 5.5175 | 1 |  |  |
|  |  |  |  |  |  |
| **(c) OTU_S** | **DF** | **Sum of Squares** | ***R^2^*** | ***F*** | ***Pr (>F)*** |
| Soil conditions (Wet vs. Mesic-dry) | 1 | 1.0027 | 0.20425 | 4.5826 | 0.001*** |
| *F. meleagris* P/A | 1 | 0.1803 | 0.03678 | 0.8241 | 0.754 |
| Residual | 17 | 3.7196 | 0.75870 |  |  |
| Total | 19 | 4.9026 | 1 |  |  |

**Table S6.** Marginal and individual PERMANOVAs of relative explanatory power of soil condition (Wet vs. Mesic-dry) and *F. meleagris* (Presence/Absence), on fungal community (a, b, c) and protists community (d, e, f) across 20 soil samples. Bray-Curtis dissimilarity index was calculated from relative abundance data from three different OTU occurrence tables (a) OTU_A, (b) OTU_C and (c) OTU_S.

| **(a) OTU_A (Fungi)** | **DF** | **Sum of Squares** | ***R^2^*** | ***F*** | ***Pr (>F)*** |
| --- | --- | --- | --- | --- | --- |
| Soil conditions (Wet vs. Mesic-dry) | 1 | 1.4360 | 0.22997 | 5.3805 | 0.001*** |
| *F. meleagris* P/A | 1 | 0.2710 | 0.04341 | 1.0155 | 0.348 |
| Residual | 17 | 4.5371 | 0.72662 |  |  |
| Total | 19 | 6.2441 | 1 |  |  |

| **(b) OTU_C (Fungi)** | **DF** | **Sum of Squares** | ***R^2^*** | ***F*** | ***Pr (>F)*** |
| --- | --- | --- | --- | --- | --- |
| Soil conditions (Wet vs. Mesic-dry) | 1 | 1.2832 | 0.25360 | 6.1320 | 0.001*** |
| *F. meleagris* P/A | 1 | 0.2193 | 0.04333 | 1.0478 | 0.287 |
| Residual | 17 | 3.5574 | 0.70307 |  |  |
| Total | 19 | 5.0598 | 1 |  |  |
| **(c) OTU_S (Fungi)** | **DF** | **Sum of Squares** | ***R^2^*** | ***F*** | ***Pr (>F)*** |
| Soil conditions (Wet vs. Mesic-dry) | 1 | 1.2147 | 0.25813 | 6.2723 | 0.001*** |
| *F. meleagris* P/A | 1 | 0.1988 | 0.04225 | 1.0267 | 0.339 |
| Residual | 17 | 3.2923 | 0.69962 |  |  |
| Total | 19 | 4.7058 | 1 |  |  |

| **(d) OTU_A (Protists)** | **DF** | **Sum of Squares** | ***R^2^*** | ***F*** | ***Pr (>F)*** |
| --- | --- | --- | --- | --- | --- |
| Soil conditions (Wet vs. Mesic-dry) | 1 | 1.4870 | 0.20660 | 4.6070 | 0.001*** |
| *F. meleagris* P/A | 1 | 0.2234 | 0.03104 | 0.6922 | 0.931 |
| Residual | 17 | 5.4869 | 0.76236 |  |  |
| Total | 19 | 7.1973 | 1 |  |  |

| **(e) OTU_C (Protists)** | **DF** | **Sum of Squares** | ***R^2^*** | ***F*** | ***Pr (>F)*** |
| --- | --- | --- | --- | --- | --- |
| Soil conditions (Wet vs. Mesic-dry) | 1 | 1.0302 | 0.17643 | 3.7923 | 0.001*** |
| *F. meleagris* P/A | 1 | 0.1909 | 0.03269 | 0.7027 | 0.932 |
| Residual | 17 | 4.6180 | 0.79088 |  |  |
| Total | 19 | 5.8391 | 1 |  |  |
|  |  |  |  |  |  |
| **(f) OTU_S (Protists)** | **DF** | **Sum of Squares** | ***R^2^*** | ***F*** | ***Pr (>F)*** |
| Soil conditions (Wet vs. Mesic-dry) | 1 | 0.7262 | 0.15127 | 3.1618 | 0.001*** |
| *F. meleagris* P/A | 1 | 0.1699 | 0.03539 | 0.7396 | 0.694 |
| Residual | 17 | 3.9045 | 0.81334 |  |  |
| Total | 19 | 4.8005 | 1 |  |  |


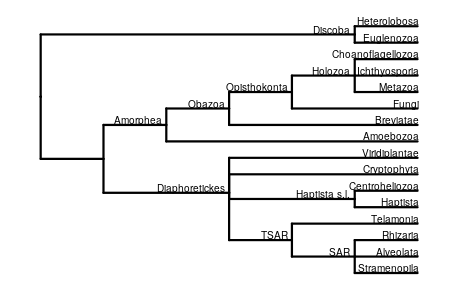


**Figure S1.** Taxonomic constraints for preliminary all-Eukaryote ML tree.


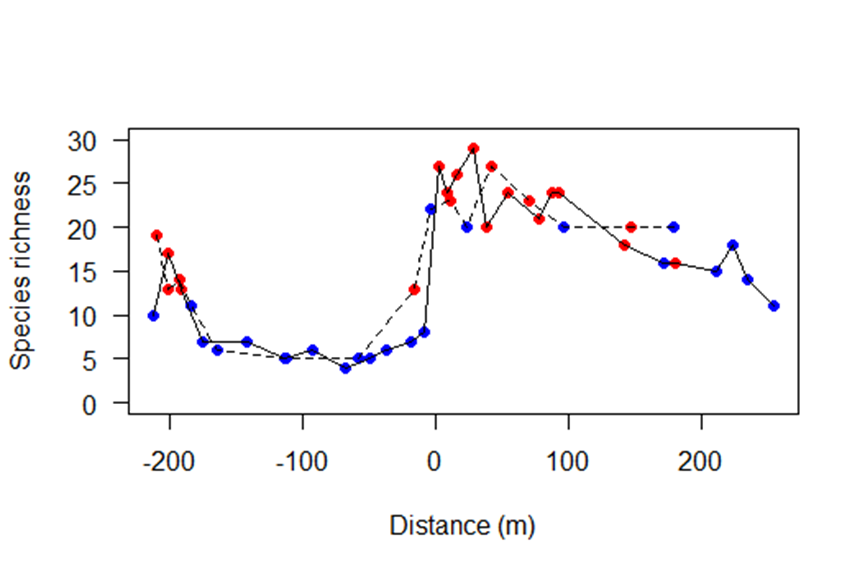


**Figure S2.** Number of plant species recorded in plots from west to east along transect 1 (continuous line) and transect 4 (dashed line). The x axis gives the distance from the soil sampling, with the wet part to the west. Red symbols indicate plots with *Fritillaria meleagris*.


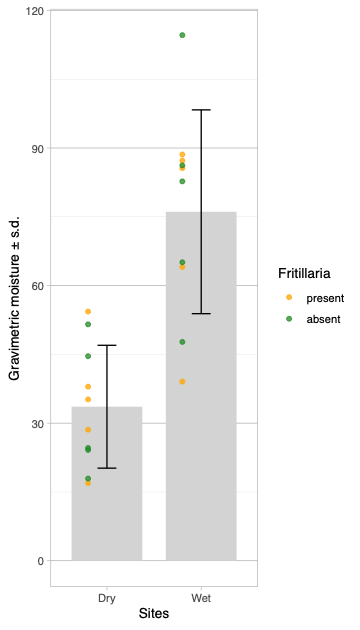


**Figure S3.** Gravimetric soil moisture in the dry and wet soil condition at Kungsängen Nature Reserve, sampled for soil community analysis. Yellow and green dots indicate soil samples taken next to Fritillaria (present) and non-Fritillaria plants (absent), respectively.


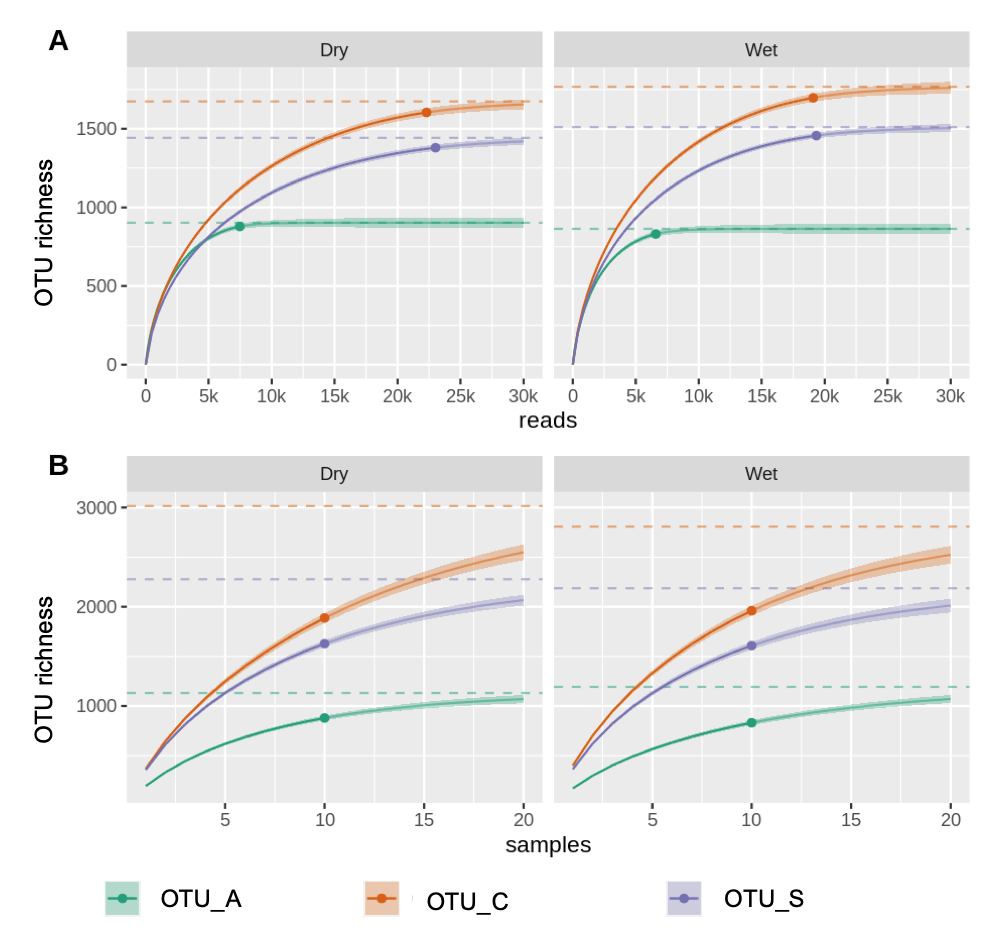


**Figure S4.** OTU accumulation curves for soil micro-eukaryotes after pooling all reads for each of the distinct plant communities associated with dry and wet soil conditions (a) based on sequencing depth and (b) based on the number of samples. Separate lines for the three OTU inference methods OTU_A in green, OTU_C in orange and OTU_S in purple. The darker line represents the accumulation curve calculated by refraction up to the dot indicating the actual total sampling followed by a lighter extrapolation line. Shaded area represents the relevant 95% intervals. Dotted horizontal lines indicate the estimated asymptotic OTU richness for each of the three OTU generation methods used.

**
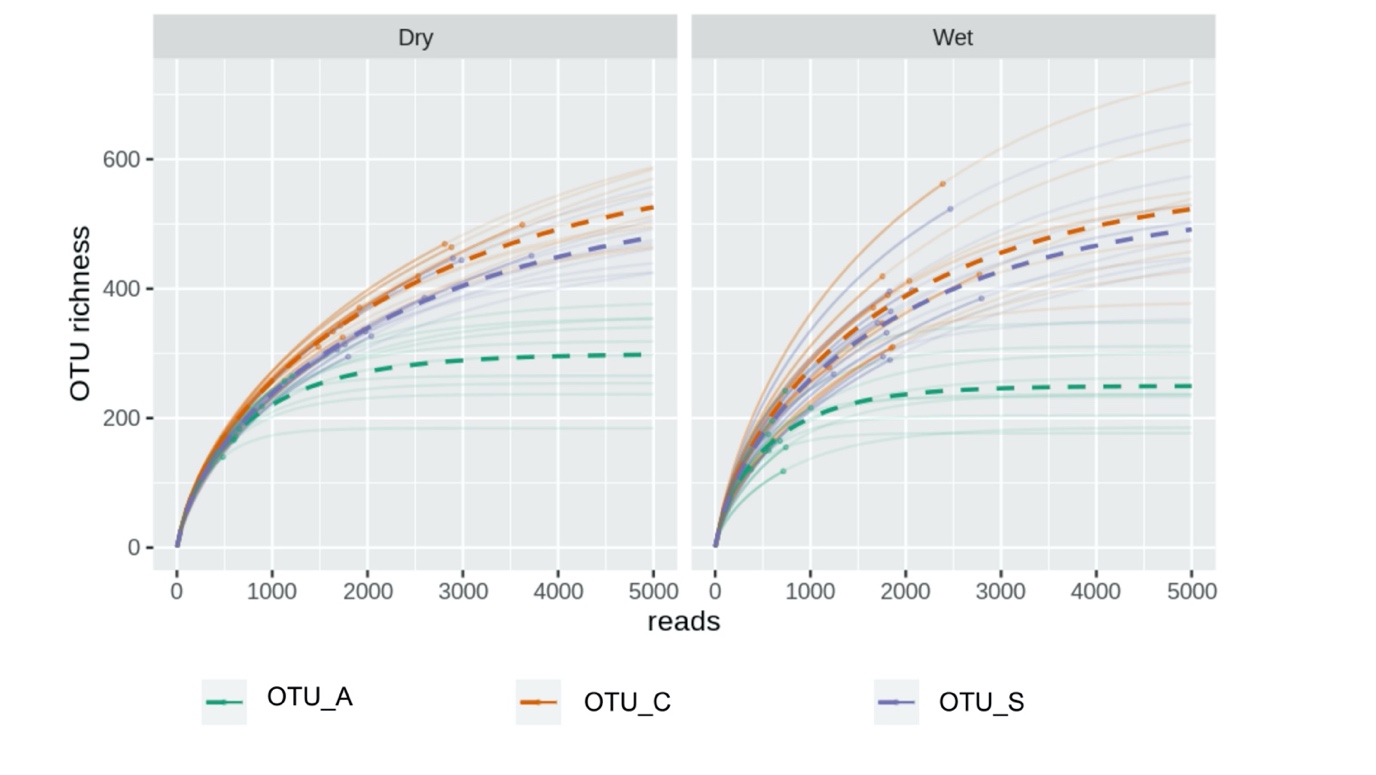
**

**Figure S5.** OTU richness curve based on reads in ten samples in dry and wet soil conditions with three different clustering methods: OTU_A (green), OTU_C (orange) and OTU_S (blue). The lines represent reads of samples in each wet and mesic-dry area. Points indicate the actual total read for each sample. Thick dashed lines represent the average for each clustering method.


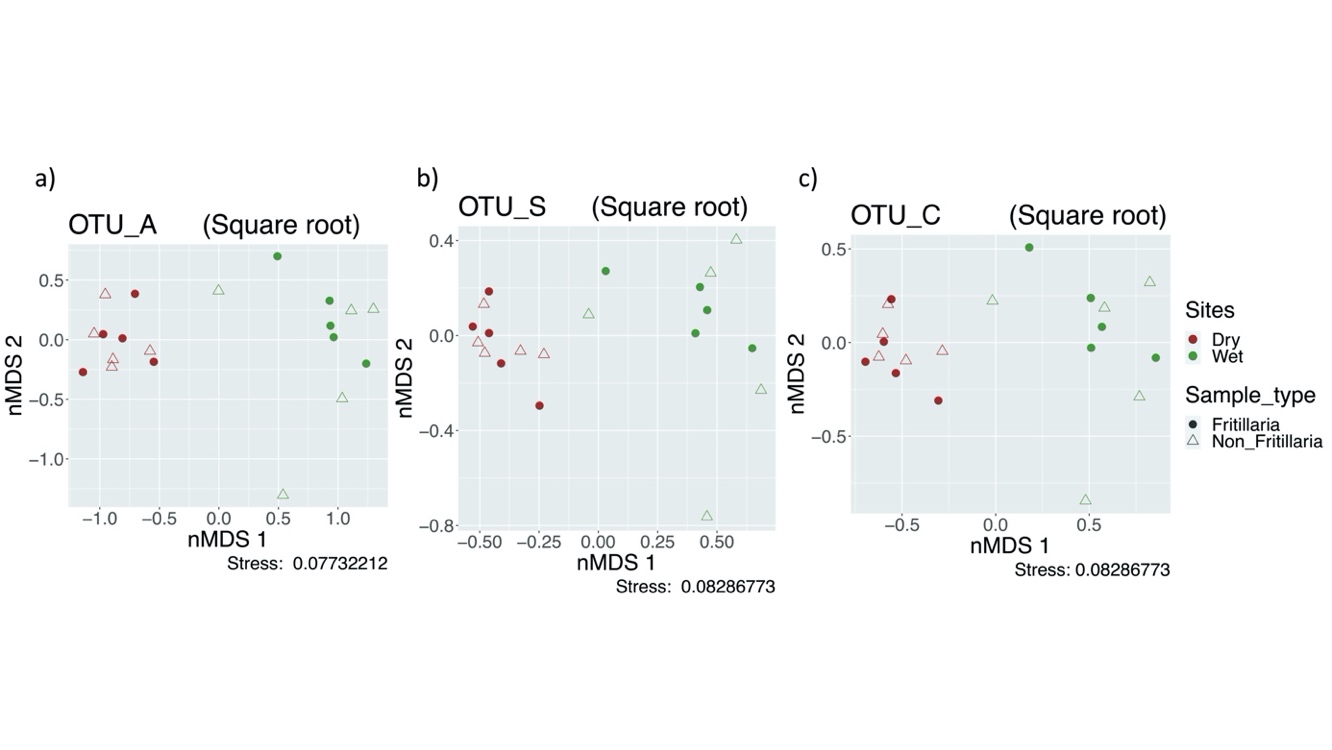


**Figure S6.** Non-metric multidimensional scaling (nMDS) ordination of soil micro-eukaryotic communities based on a Bray-Curtis dissimilarity index applied to square root transformed relative abundances of the three different OTU inference methods, a) OTU_A , b) OTU_S and c) OTU_C. Red and green indicate soil samples in mesic-dry and wet soil condition respectively and circle and triangle indicate samples with or without *F. meleagris*.


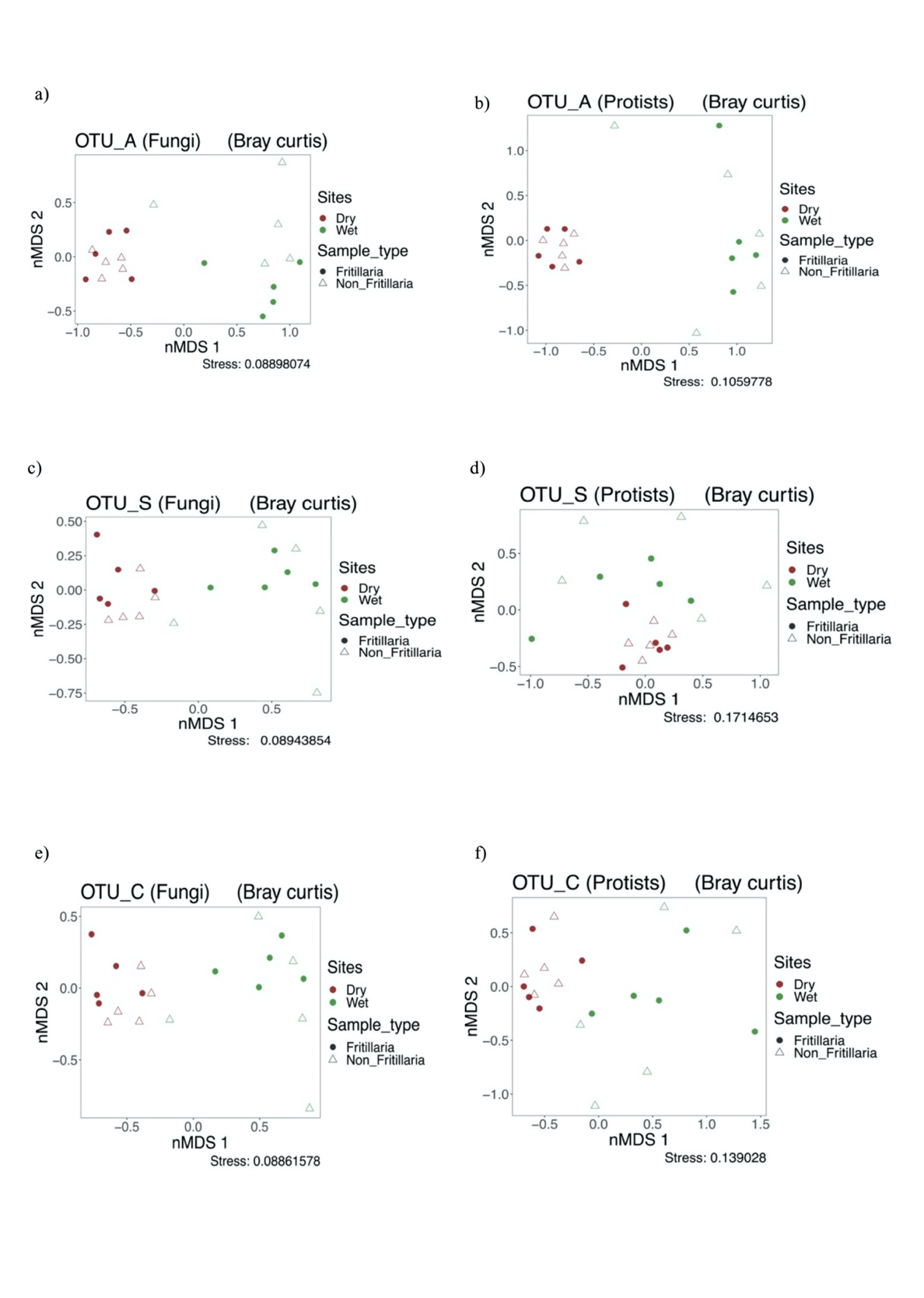


**Figure S7.** Non-metric multidimensional scaling (nMDS) ordination of soil micro-eukaryotic community based on a Bray-Curtis dissimilarity index applied to relative abundances of the three different OTU inference methods, OTU_A, OTU_S and OTU_C. Analysed separately into a, c, e) fungi and b, d, f) protists. Red and green indicate samples in mesic-dry and wet soil condition respectively and circle and triangle indicate samples with or without *F. meleagris*.


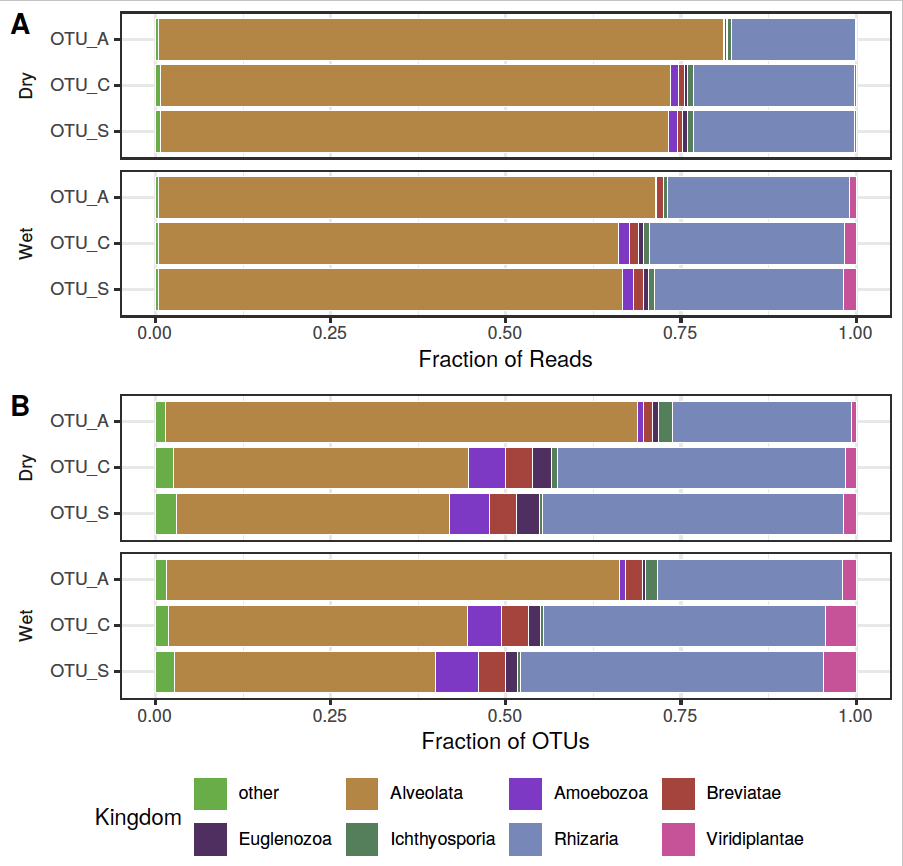


**Figure S8.** Kingdom-level taxonomic assignment of protists, in wet and mesic-dry conditions for the three occurrence tables OTU_A, OTU_C and OTU_S, A) Mean fractional read abundance; B) Fractional OTU_A richness. Kingdoms which represents less than 1.5% of total OTUs and less than 1.5% of total reads are grouped together as “other”.  (Kingdoms *sensu* Tedersoo 2017).


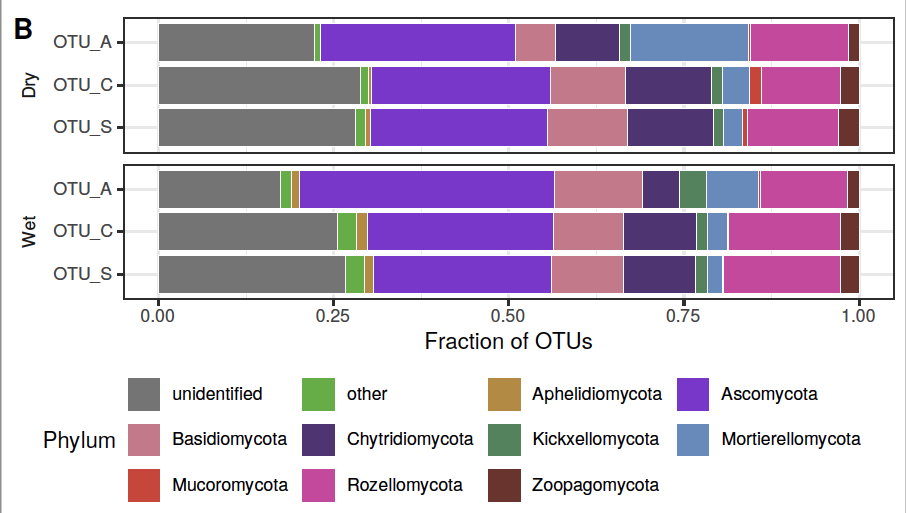


**Figure S9.** Phylum-level taxonomic assignment of fungi at wet and mesic-dry sampling locations as fraction of OTU_A richness. Phyla which represents less than 1.5% of total OTUs and less than 1.5% of total reads are grouped together as “other”.


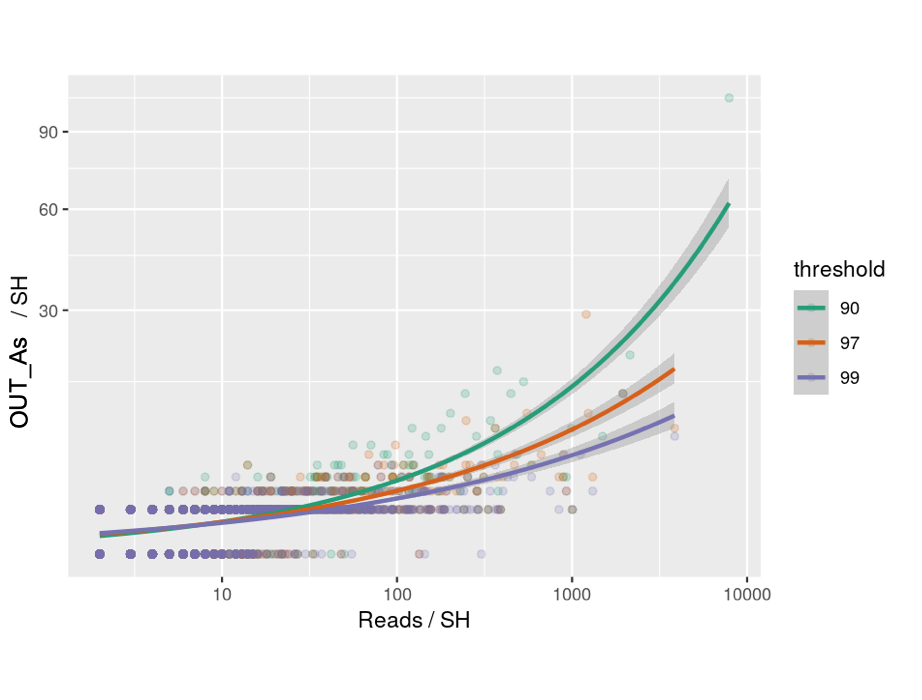


**Figure S10.** Number of OTU_As recovered per species hypothesis (SH) depending on number of reads per SH, for the three ITS sequence similarity thresholds 90% (green), 97% (red) and 99% (purple).


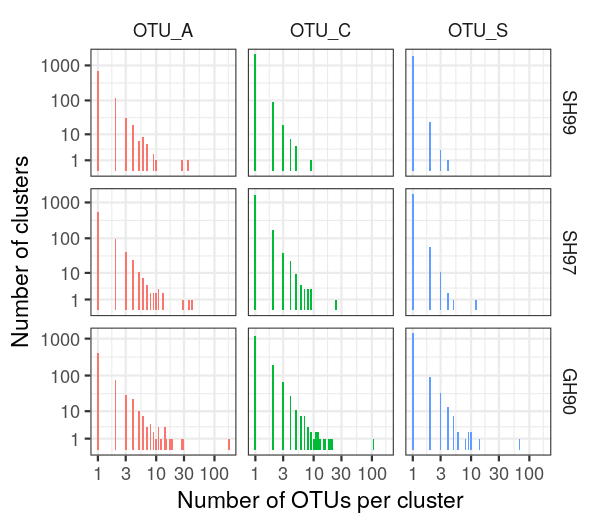


**Figure S11.** Number of species hypothesis clusters (SH_99, SH_97 and GH_90) represented by different number of OTUs separately for the three different OTU inference methods OTU_A, OTU_C and OTU_S.

**
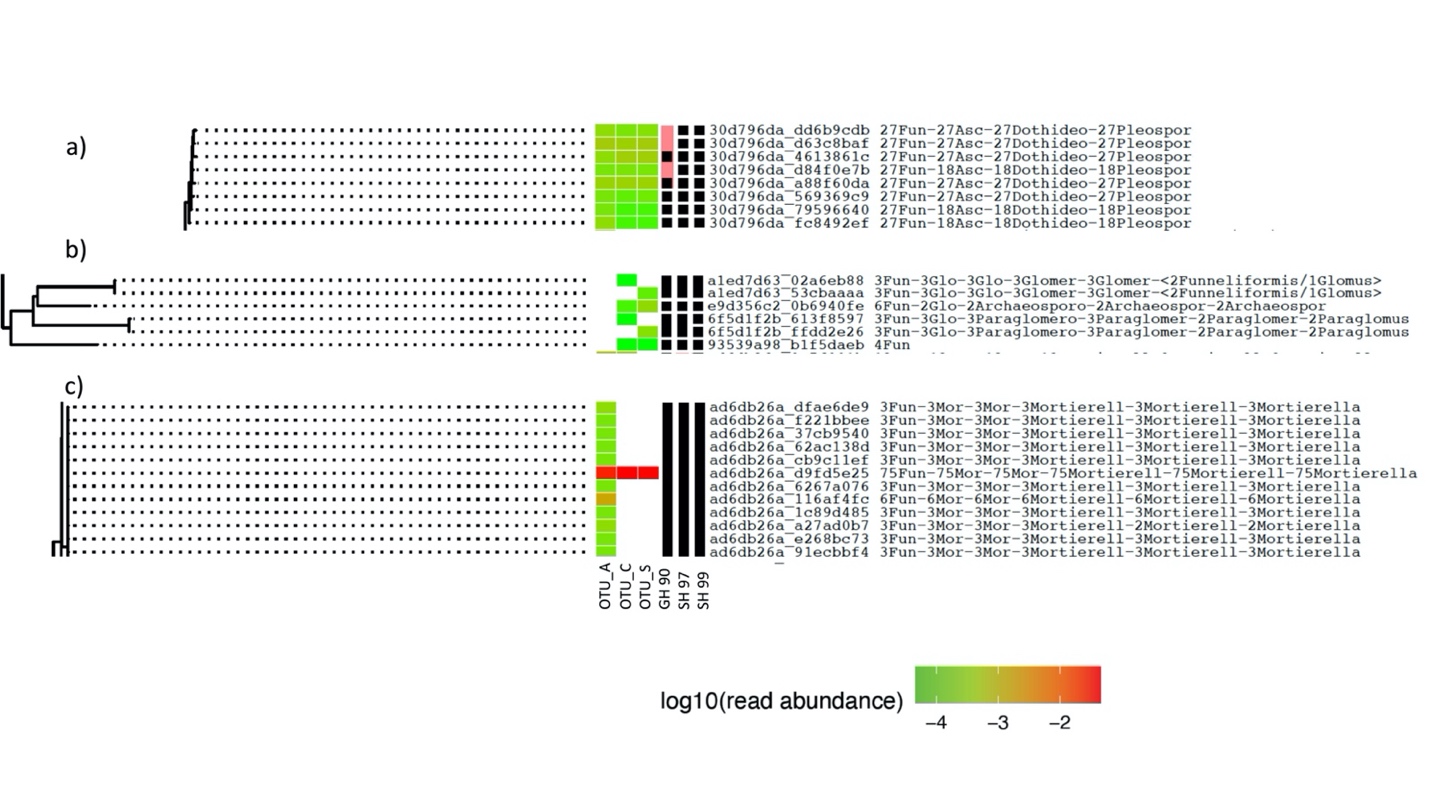
Figure S12**. Phylogenetic sister lineages with sequences from the three OTU methods (three colored boxes from left to right: OTU_A, OTU_C and OTU_S; green represents low abundance, and red represents high abundance), clustered based on sequence similarity over the ITS2 region (black boxes/strips from left to right GH_90, SH_97 and SH_99, red when polyphyletic). a) Examples of polyphyletic GH_90, with short branch lengths separating potential species in SH_97 and SH_99. b) Rare taxa are often not represented at all by OTU_A sequences as in this case for Glomeromycota. c) in abundant taxa on the other hand OTU_A captures a high level of intra species genetic variation that is collapsed into one or two sequence variants in OTU_C and OTU_S as in this case of one *Morteriella* SH_99 represented by 12 OTU_A sequences.

**Figure S13.** Number of OTU_Ss assigned to a) fungi and b) protists, as detected across mesic-dry and wet soil samples. c) Relative abundance of both fungi and protists, detected in samples on the mesic-dry (left) and wet (right) side, OTU_Ss found only on either mesic-dry or wet side is displayed separately from those common to both types of samples from the studied soil moisture transition.

**Figure S14.** In total 15 micro-eukaryotic OTU_Ss that are differentially abundant in wet vs mesic-dry condition based on DESeq analysis. These OTUs belong to the three kingdom, Rhizaria (red), Fungi (green) and Alveolata (blue) and five phyla.


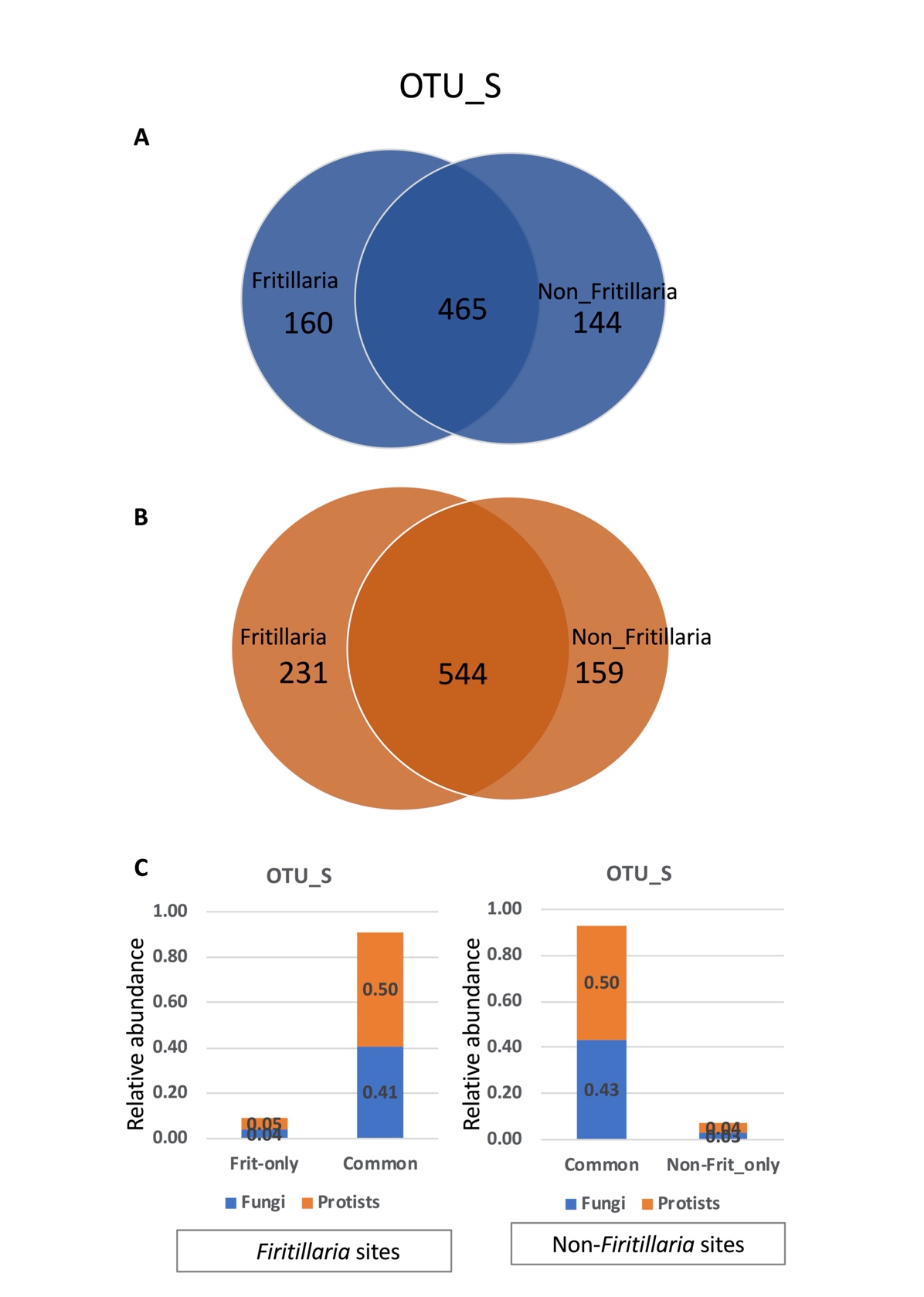


**Figure S15.** Number of OTU_Ss assigned to a) fungi and b) protists, as detected across *F. meleagris* and non- *F. meleagris* soil samples. c) Relative abundance of both fungi and protists, detected in *F. meleagris* (left) and non- *F. meleagris* (right) samples, OTU_As found only in either *F. meleagris* and non- *F. meleagris* is displayed separately from those common to both types of samples.
