# Supplementary figures and images for: Effects of operational taxonomic unit inference methods on soil microeukaryote community analysis using long‐read metabarcoding
Source: Ecol Evol. 2022 Mar 8;12(3):e8676. doi: 10.1002/ece3.8676 (PMC8928899; doi:10.1002/ece3.8676)

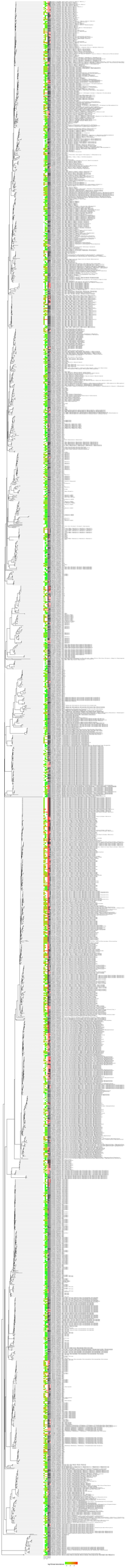

Supplement: Supplementary file 3 — Data S2 [file ECE3-12-e8676-s006.pdf]

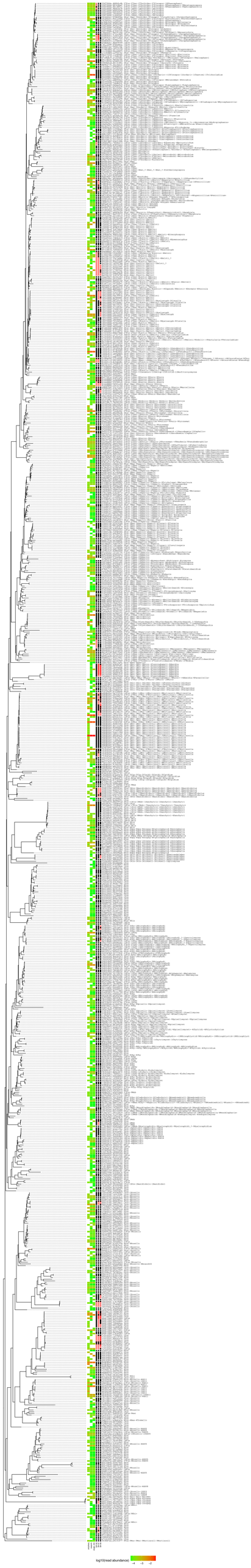

Supplement: Supplementary file 4 — Data S3 [file ECE3-12-e8676-s003.pdf]
